# Supplementary material for: A systematic review on improving implementation of the revitalised integrated disease surveillance and response system in the African region: A health workers’ perspective
Source: PLoS One. 2021 Mar 19;16(3):e0248998. doi: 10.1371/journal.pone.0248998 (PMC7978283; doi:10.1371/journal.pone.0248998)
Supplement: S1 Table — (DOCX) [file pone.0248998.s001.docx]

**S1 Table. Excluded studies from systematic literature review**

| **No.** | **Reasons for exclusion of studies** | **Description of excluded studies** |
| --- | --- | --- |
| 1. | A | Abakar MF, Schelling E, Bechir M, Ngandolo BN, Pfister K, Alfaroukh IO, et al. Trends in health surveillance and joint service delivery for pastoralists in West and Central Africa. Rev Sci Tech. 2016;35(2):683-91. Epub 2016/12/06. doi: 10.20506/rst.35.2.2549. PubMed PMID: 27917961. |
| 2. | A | Abass KM, van der Werf TS, Phillips RO, Sarfo FS, Abotsi J, Mireku SO, et al. Buruli ulcer control in a highly endemic district in Ghana: role of community-based surveillance volunteers. Am J Trop Med Hyg. 2015;92(1):115-7. Epub 2014/10/22. doi: 10.4269/ajtmh.14-0405. PubMed PMID: 25331802; PubMed Central PMCID: PMCPMC4347364. |
| 3. | A | Abdel-Mannan OA, Harris MJ, Parker JA, Aly GS, El-Sayed NM. Testing clinical surveillance of acute flaccid paralysis in Egypt post-eradication of poliomyelitis. Trop Med Int Health. 2010;15(11):1395-400. Epub 2010/10/20. doi: 10.1111/j.1365-3156.2010.02636.x. PubMed PMID: 20955500. |
| 4. | A | Abdel-Wahab F, Ghoneim M, Khashaba M, El-Gilany AH, Abdel-Hady D. Nosocomial infection surveillance in an Egyptian neonatal intensive care unit. J Hosp Infect. 2013;83(3):196-9. Epub 2013/02/05. doi: 10.1016/j.jhin.2012.10.017. PubMed PMID: 23374289. |
| 5. | A | Abebe SM, Andargie G, Shimeka A, Alemu K, Kebede Y, Wubeshet M, et al. The prevalence of non-communicable diseases in northwest Ethiopia: survey of Dabat Health and Demographic Surveillance System. BMJ Open. 2017;7(10):e015496. Epub 2017/10/25. doi: 10.1136/bmjopen-2016-015496. PubMed PMID: 29061601; PubMed Central PMCID: PMCPMC5665308. |
| 6. | A | Abouchadi S, Zhang WH, De Brouwere V. Underreporting of deaths in the maternal deaths surveillance system in one region of Morocco. PLoS One. 2018;13(1):e0188070. Epub 2018/02/01. doi: 10.1371/journal.pone.0188070. PubMed PMID: 29385140; PubMed Central PMCID: PMCPMC5791944. |
| 7. | A | AC CC, Codeço CT, Honório NA, Pereira GR, CF NP, Nobre AA. Surveillance of dengue vectors using spatio-temporal Bayesian modeling. BMC Med Inform Decis Mak. 2015;15:93. Epub 2015/11/17. doi: 10.1186/s12911-015-0219-6. PubMed PMID: 26566610; PubMed Central PMCID: PMCPMC4644323. |
| 8. | A | Acup C, Bardosh KL, Picozzi K, Waiswa C, Welburn SC. Factors influencing passive surveillance for T. b. rhodesiense human african trypanosomiasis in Uganda. Acta Trop. 2017;165:230-9. Epub 2016/05/24. doi: 10.1016/j.actatropica.2016.05.009. PubMed PMID: 27212706. |
| 9. | A | Adegoke OJ, Takane M, Biya O, Ota M, Murele B, Mahoney F, et al. Strengthening the Acute Flaccid Paralysis (AFP) Surveillance Component of the Polio Eradication Initiative through Short Message Service (SMS) Reminders; Experience from Sokoto State, Nigeria 2014. Journal of immunological sciences. 2018;Suppl(10):68-74. Epub 2019/03/08. PubMed PMID: 30842999; PubMed Central PMCID: PMCPMC6398576. |
| 10. | A | Adomako J, Asare GQ, Ofosu A, Iott BE, Anthony T, Momoh AS, et al. Community-based surveillance of maternal deaths in rural Ghana. Bull World Health Organ. 2016;94(2):86-91. Epub 2016/02/26. doi: 10.2471/blt.15.154849. PubMed PMID: 26908958; PubMed Central PMCID: PMCPMC4750432. |
| 11. | A | Adongo PB, Tabong PT, Asampong E, Ansong J, Robalo M, Adanu RM. Health workers perceptions and attitude about Ghana's preparedness towards preventing, containing, and managing Ebola Virus Disease. BMC Health Serv Res. 2017;17(1):266. Epub 2017/04/14. doi: 10.1186/s12913-017-2225-0. PubMed PMID: 28403852; PubMed Central PMCID: PMCPMC5389014. |
| 12. | A | Afework MF, Gebregiorgis SH, Roro MA, Lemma AM, Ahmed S. Do Health and Demographic Surveillance Systems benefit local populations? Maternal care utilisation in Butajira HDSS, Ethiopia. Global health action. 2014;7:24228. Epub 2014/07/08. doi: 10.3402/gha.v7.24228. PubMed PMID: 24998383; PubMed Central PMCID: PMCPMC4083147. |
| 13. | A | Agier L, Broutin H, Bertherat E, Djingarey MH, Lingani C, Perea W, et al. Timely detection of bacterial meningitis epidemics at district level: a study in three countries of the African Meningitis Belt. Trans R Soc Trop Med Hyg. 2013;107(1):30-6. Epub 2013/01/09. doi: 10.1093/trstmh/trs010. PubMed PMID: 23296695. |
| 14. | A | Alabi O, Doctor HV, Afenyadu GY, Findley SE. Lessons learned from setting up the Nahuche Health and Demographic Surveillance System in the resource-constrained context of northern Nigeria. Global health action. 2014;7:23368. Epub 2014/05/09. doi: 10.3402/gha.v7.23368. PubMed PMID: 24809831; PubMed Central PMCID: PMCPMC4014660. |
| 15. | A | Alleman MM, Meyer SA, Mulumba A, Nyembwe M, Riziki Y, Mbule A, et al. Improved acute flaccid paralysis surveillance performance in the Democratic Republic of the Congo, 2010-2012. J Infect Dis. 2014;210 Suppl 1:S50-61. Epub 2014/10/16. doi: 10.1093/infdis/jit670. PubMed PMID: 25316874. |
| 16. | A | Amek N, Vounatsou P, Obonyo B, Hamel M, Odhiambo F, Slutsker L, et al. Using health and demographic surveillance system (HDSS) data to analyze geographical distribution of socio-economic status; an experience from KEMRI/CDC HDSS. Acta Trop. 2015;144:24-30. Epub 2015/01/21. doi: 10.1016/j.actatropica.2015.01.006. PubMed PMID: 25602533. |
| 17. | A | Ameme DK, Nyarko KM, Kenu E, Afari EA. Strengthening surveillance and response to public health emergencies in the West African sub-region: the role of Ghana FELTP. Pan Afr Med J. 2016;25(Suppl 1):1. Epub 2017/02/06. doi: 10.11604/pamj.supp.2016.25.1.10579. PubMed PMID: 28149432; PubMed Central PMCID: PMCPMC5257012. |
| 18. | A | Amouzou A, Kanyuka M, Hazel E, Heidkamp R, Marsh A, Mleme T, et al. Independent Evaluation of the integrated Community Case Management of Childhood Illness Strategy in Malawi Using a National Evaluation Platform Design. Am J Trop Med Hyg. 2016;94(3):574-83. Epub 2016/01/21. doi: 10.4269/ajtmh.15-0584. PubMed PMID: 26787158; PubMed Central PMCID: PMCPMC4775894. |
| 19. | A | Amukele T. Africa CDC: Establishing Integrated Surveillance and Laboratory Networks for Rapid Disease Detection and Response, Control, Prevention, and Clinical Care in Africa. African journal of laboratory medicine. 2017;6(1):638. Epub 2017/09/08. doi: 10.4102/ajlm.v6i1.638. PubMed PMID: 28879156; PubMed Central PMCID: PMCPMC5523921. |
| 20. | A | Andre AM, Lopez A, Perkins S, Lambert S, Chace L, Noudeke N, et al. Frontline Field Epidemiology Training Programs as a Strategy to Improve Disease Surveillance and Response. Emerg Infect Dis. 2017;23(13). Epub 2017/11/21. doi: 10.3201/eid2313.170803. PubMed PMID: 29155657; PubMed Central PMCID: PMCPMC5711307. |
| 21. | A | Andriamandimby SF, Heraud JM, Ramiandrasoa R, Ratsitorahina M, Rasambainarivo JH, Dacheux L, et al. Surveillance and control of rabies in La Reunion, Mayotte, and Madagascar. Vet Res. 2013;44. doi: 10.1186/1297-9716-44-77. PubMed PMID: WOS:000324856200001. |
| 22. | A | Ashenafi W, Eshetu F, Assefa N, Oljira L, Dedefo M, Zelalem D, et al. Trend and causes of adult mortality in Kersa health and demographic surveillance system (Kersa HDSS), eastern Ethiopia: verbal autopsy method. Population health metrics. 2017;15(1):22. Epub 2017/07/02. doi: 10.1186/s12963-017-0144-2. PubMed PMID: 28666480; PubMed Central PMCID: PMCPMC5493878. |
| 23. | B | Awini EA, Bonney JHK, Frimpong JA, Ampofo WK, Koram KA. Information gaps in surveillance data and effects on the Ghanaian response to the Ebola outbreak in West Africa. Ghana Med J. 2017;51(3):115-9. Epub 2018/04/07. PubMed PMID: 29622822; PubMed Central PMCID: PMCPMC5870230. |
| 24. | A | Ayede A, Bello FA, Kehinde AO. A Community-based Surveillance of Gastrointestinal Helminthiasis among Pregnant Women in Ibadan, South West Nigeria. Niger J Clin Pract. 2018;21(10):1368-73. doi: 10.4103/njcp.njcp_196_17. PubMed PMID: WOS:000446795500021. |
| 25. | A | Ayeh-Kumi PF, Obeng-Nkrumah N, Baidoo D, Teye J, Asmah RH. High levels of urinary schistosomiasis among children in Bunuso, a rural community in Ghana: an urgent call for increased surveillance and control programs. J Parasit Dis. 2015;39(4):613-23. Epub 2015/12/22. doi: 10.1007/s12639-013-0411-5. PubMed PMID: 26688622; PubMed Central PMCID: PMCPMC4675572. |
| 26. | B | Ayele W, Demissie G, Kassa W, Zemelak E, Afework A, Amare B, et al. Challenges of establishing routine influenza sentinel surveillance in Ethiopia, 2008-2010. J Infect Dis. 2012;206 Suppl 1:S41-5. Epub 2012/11/28. doi: 10.1093/infdis/jis531. PubMed PMID: 23169970. |
| 27. | A | Beguy D, Elung'ata P, Mberu B, Oduor C, Wamukoya M, Nganyi B, et al. Health & Demographic Surveillance System Profile: The Nairobi Urban Health and Demographic Surveillance System (NUHDSS). Int J Epidemiol. 2015;44(2):462-71. Epub 2015/01/18. doi: 10.1093/ije/dyu251. PubMed PMID: 25596586. |
| 28. | A | Benson FG, Levin J, Rispel LC. Health care providers' compliance with the notifiable diseases surveillance system in South Africa. PLoS One. 2018;13(4):e0195194. Epub 2018/04/10. doi: 10.1371/journal.pone.0195194. PubMed PMID: 29630627; PubMed Central PMCID: PMCPMC5891014. |
| 29. | A | Biggs HM, Hertz JT, Munishi OM, Galloway RL, Marks F, Saganda W, et al. Estimating leptospirosis incidence using hospital-based surveillance and a population-based health care utilization survey in Tanzania. PLoS Negl Trop Dis. 2013;7(12):e2589. Epub 2013/12/18. doi: 10.1371/journal.pntd.0002589. PubMed PMID: 24340122; PubMed Central PMCID: PMCPMC3855074. |
| 30. | A | Broban A, Tejiokem MC, Tiembré I, Druelles S, L'Azou M. Bolstering human rabies surveillance in Africa is crucial to eliminating canine-mediated rabies. PLoS Negl Trop Dis. 2018;12(9):e0006367. Epub 2018/09/07. doi: 10.1371/journal.pntd.0006367. PubMed PMID: 30188896; PubMed Central PMCID: PMCPMC6126826 following competing interests: AB was employed by the funder under temporary contract during the study. ML and SD are employees of the funder, Sanofi Pasteur. The other authors declare that no competing interests exist. |
| 31. | A | Budgell E, Cohen AL, McAnerney J, Walaza S, Madhi SA, Blumberg L, et al. Evaluation of two influenza surveillance systems in South Africa. PLoS One. 2015;10(3):e0120226. Epub 2015/03/31. doi: 10.1371/journal.pone.0120226. PubMed PMID: 25822719; PubMed Central PMCID: PMCPMC4379032. |
| 32. | A | Bwire G, Ali M, Sack DA, Nakinsige A, Naigaga M, Debes AK, et al. Identifying cholera "hotspots" in Uganda: An analysis of cholera surveillance data from 2011 to 2016. PLoS Negl Trop Dis. 2017;11(12):e0006118. Epub 2017/12/29. doi: A 10.1371/journal.pntd.0006118. PubMed PMID: 29284003; PubMed A Central PMCID: PMCPMC5746206. |
| 33. | B | Caceres VM, Sidibe S, Andre M, Traicoff D, Lambert S, King M, et al. Surveillance Training for Ebola Preparedness in Cote d'Ivoire, Guinea-Bissau, Senegal, and Mali. Emerg Infect Dis. 2017;23(13). Epub 2017/11/21. doi: 10.3201/eid2313.170299. PubMed PMID: 29155654; PubMed Central PMCID: PMCPMC5711303. |
| 34. | A | Chen Y, Han L, Liu C. Current status of surveillance for infectious diseases in Africa. Zhonghua liu xing bing xue za zhi = Zhonghua liuxingbingxue zazhi. 2018;39(11):1530-4. Epub 2018/11/22. doi: 10.3760/cma.j.issn.0254-6450.2018.11.021. PubMed PMID: 30462967. |
| 35. | B | Chisha Z, Larsen DA, Burns M, Miller JM, Chirwa J, Mbwili C, et al. Enhanced surveillance and data feedback loop associated with improved malaria data in Lusaka, Zambia. Malar J. 2015;14:222. Epub 2015/05/29. doi: 10.1186/s12936-015-0735-y. PubMed PMID: 26017275; PubMed Central PMCID: PMCPMC4486393. |
| 36. | A | Date K, Person B, Nygren B, Were V, Kola S, Ayers T, et al. Evaluation of a Rapid Cholera Response Activity-Nyanza Province, Kenya, 2008. J Infect Dis. 2013;208(suppl_1) Supplement(1):S62-S8. |
| 37. | A | Di Pasquale A, McCann RS, Maire N. Assessing the population coverage of a health demographic surveillance system using satellite imagery and crowd-sourcing. PLoS One. 2017;12(8):e0183661. Epub 2017/09/01. doi: 10.1371/journal.pone.0183661. PubMed PMID: 28859109; PubMed Central PMCID: PMCPMC5578500. |
| 38. | A | Diese M, Kalonji A, Izale B, Villeneuve S, Kintaudi NM, Clarysse G, et al. Community-based maternal, newborn, and child health surveillance: perceptions and attitudes of local stakeholders towards using mobile phone by village health volunteers in the Kenge Health Zone, Democratic Republic of Congo. BMC Public Health. 2018;18(1):316. Epub 2018/03/07. doi: 10.1186/s12889-018-5186-2. PubMed PMID: 29506500; PubMed Central PMCID: PMCPMC5838964. |
| 39. | A | Dil Y, Strachan D, Cairncross S, Korkor AS, Hill Z. Motivations and challenges of community-based surveillance volunteers in the northern region of Ghana. J Community Health. 2012;37(6):1192-8. Epub 2012/05/23. doi: 10.1007/s10900-012-9569-5. PubMed PMID: 22614535. |
| 40. | A | Diomidous M, Pistolis J, Mechili A, Kolokathi A, Zimeras S. Healthcare Information Systems for the epidemiologic surveillance within the community. Stud Health Technol Inform. 2013;190:252-4. Epub 2013/07/05. PubMed PMID: 23823439. |
| 41. | B | Djomassi LD, Gessner BD, Andze GO, Mballa GA. National surveillance data on the epidemiology of cholera in Cameroon. J Infect Dis. 2013;208 Suppl 1:S92-7. Epub 2013/10/23. doi: 10.1093/infdis/jit197. PubMed PMID: 24101652. |
| 42. | B | Doctor SM, Liu Y, Whitesell A, Thwai KL, Taylor SM, Janko M, et al. Malaria surveillance in the Democratic Republic of the Congo: comparison of microscopy, PCR, and rapid diagnostic test. Diagn Microbiol Infect Dis. 2016;85(1):16-8. Epub 2016/02/27. doi: 10.1016/j.diagmicrobio.2016.01.004. PubMed PMID: 26915637; PubMed Central PMCID: PMCPMC4841725. |
| 43. | A | Dorkenoo MA, Barrette A, Agbo YM, Bogreau H, Kutoati S, Sodahlon YK, et al. Surveillance of the efficacy of artemether-lumefantrine and artesunate-amodiaquine for the treatment of uncomplicated Plasmodium falciparum among children under five in Togo, 2005-2009. Malar J. 2012;11:338. Epub 2012/10/10. doi: 10.1186/1475-2875-11-338. PubMed PMID: 23043495; PubMed Central PMCID: PMCPMC3507743. |
| 44. | A | Dorkenoo MA, Bronzan R, Yehadji D, Tchalim M, Yakpa K, Etassoli S, et al. Surveillance for lymphatic filariasis after stopping mass drug administration in endemic districts of Togo, 2010-2015. Parasites & vectors. 2018;11(1):244. Epub 2018/04/18. doi: 10.1186/s13071-018-2843-3. PubMed PMID: 29661231; PubMed Central PMCID: PMCPMC5902853. |
| 45. | A | Dzotsi EK, Dongdem AZ, Boateng G, Antwi L, Owusu-Okyere G, Nartey DB, et al. Surveillance of Bacterial Pathogens of Diarrhoea in Two Selected Sub Metros Within the Accra Metropolis. Ghana Med J. 2015;49(2):65-71. Epub 2015/09/05. doi: 10.4314/gmj.v49i2.1. PubMed PMID: 26339088; PubMed Central PMCID: PMCPMC4549830. |
| 46. | A | Ebile AW, Ateudjieu J, Yakum MN, Djuidje MN, Watcho P. Assessing the detection, reporting and investigation of adverse events in clinical trial protocols implemented in Cameroon: a documentary review of clinical trial protocols. BMC Med Ethics. 2015;16(1):67. Epub 2015/10/01. doi: 10.1186/s12910-015-0061-5. PubMed PMID: 26420169; PubMed Central PMCID: PMCPMC4589194. |
| 47. | B | Ernst KC. Measles surveillance in Nigeria: enough information for policy making? Trans R Soc Trop Med Hyg. 2014;108(6):311-2. Epub 2014/04/30. doi: 10.1093/trstmh/tru060. PubMed PMID: 24778206. |
| 48. | A | Fatiregun AA, Adebowale AS, Fagbamigbe AF. Epidemiology of measles in Southwest Nigeria: an analysis of measles case-based surveillance data from 2007 to 2012. Trans R Soc Trop Med Hyg. 2014;108(3):133-40. Epub 2014/01/30. doi: 10.1093/trstmh/tru004. PubMed PMID: 24473475. |
| 49. | B | Frimpong JA, Amo-Addae MP, Adewuyi PA, Hall CD, Park MM, Nagbe TK. Detecting, reporting, and analysis of priority diseases for routine public health surveillance in Liberia. Pan Afr Med J. 2017;27(Suppl 1):10. Epub 2017/07/20. doi: 10.11604/pamj.supp.2017.27.1.12561. PubMed PMID: 28721174; PubMed Central PMCID: PMCPMC5500932. |
| 50. | B | Frimpong JA, Amo-Addae MP, Adewuyi PA, Hall CD, Park MM, Nagbe TK. Conducting a surveillance problem analysis on poor feedback from Reference Laboratory, Liberia, February 2016. Pan Afr Med J. 2017;27(Suppl 1):11. Epub 2017/07/20. doi: 10.11604/pamj.supp.2017.27.1.12569. PubMed PMID: 28721175; PubMed Central PMCID: PMCPMC5500934. |
| 51. | A | Fuller TL, Ducatez MF, Njabo KY, Couacy-Hymann E, Chasar A, Aplogan GL, et al. Avian influenza surveillance in Central and West Africa, 2010-2014. Epidemiol Infect. 2015;143(10):2205-12. Epub 2014/12/23. doi: 10.1017/s0950268814003586. PubMed PMID: 25530320. |
| 52. | B | Girdler-Brown BV. Evaluation of the notifiable diseases surveillance system in South Africa. Int J Infect Dis. 2017;59:139-40. Epub 2017/05/23. doi: 10.1016/j.ijid.2017.03.022. PubMed PMID: 28528937. |
| 53. | A | Green JL, Reynolds KM, Banner W, Bond GR, Kauffman RE, Palmer RB, et al. Evaluation of the quality and value of data sources for postmarket surveillance of the safety of cough and cold medications in children. BMC Med Res Methodol. 2018;18(1):175. Epub 2018/12/24. doi: 10.1186/s12874-018-0626-3. PubMed PMID: 30577764; PubMed Central PMCID: PMCPMC6303866. |
| 54. | A | Gujral L, Sema C, Rebaudet S, Taibo CL, Manjate AA, Piarroux R, et al. Cholera epidemiology in Mozambique using national surveillance data. J Infect Dis. 2013;208 Suppl 1:S107-14. Epub 2013/10/23. doi: 10.1093/infdis/jit212. PubMed PMID: 24101638. |
| 55. | A | Homan T, di Pasquale A, Onoka K, Kiche I, Hiscox A, Mweresa C, et al. Profile: The Rusinga Health and Demographic Surveillance System, Western Kenya. Int J Epidemiol. 2016;45(3):718-27. Epub 2016/05/18. doi: 10.1093/ije/dyw072. PubMed PMID: 27185811. |
| 56. | A | Houlihan CF, Youkee D, Brown CS. Novel surveillance methods for the control of Ebola virus disease. International health. 2017;9(3):139-41. Epub 2017/06/06. doi: 10.1093/inthealth/ihx010. PubMed PMID: 28582554. |
| 57. | A | Jephcott FL, Wood JLN, Cunningham AA. Facility-based surveillance for emerging infectious diseases; diagnostic practices in rural west african hospital settings: Observations from Ghana. Philosophical Transactions of the Royal Society B: Biological Sciences. 2017;372(1725). doi: 10.1098/rstb.2016.0544. |
| 58. | A | Jephcott FL, Wood JLN, Cunningham AA. Facility-based surveillance for emerging infectious diseases; diagnostic practices in rural West African hospital settings: observations from Ghana. Philos Trans R Soc Lond B Biol Sci. 2017;372(1725). Epub 2017/06/07. doi: 10.1098/rstb.2016.0544. PubMed PMID: 28584181; PubMed Central PMCID: PMCPMC5468698. |
| 59. | A | Jia K, Mohamed K. Evaluating the use of cell phone messaging for community Ebola syndromic surveillance in high risked settings in Southern Sierra Leone. Afr Health Sci. 2015;15(3):797-802. Epub 2016/03/10. doi: 10.4314/ahs.v15i3.13. PubMed PMID: 26957967; PubMed Central PMCID: PMCPMC4765450. |
| 60. | B | Jima D, Wondabeku M, Alemu A, Teferra A, Awel N, Deressa W, et al. Analysis of malaria surveillance data in Ethiopia: what can be learned from the Integrated Disease Surveillance and Response System? Malar J. 2012;11:330. Epub 2012/09/19. doi: 10.1186/1475-2875-11-330. PubMed PMID: 22985409; PubMed Central PMCID: PMCPMC3528460. |
| 61. | A | Joshi A, Amadi C, Trout K, Obaro S. Evaluation of an interactive surveillance system for monitoring acute bacterial infections in Nigeria. Perspectives in health information management. 2014;11:1f. Epub 2014/05/09. PubMed PMID: 24808807; PubMed Central PMCID: PMCPMC3995493. |
| 62. | B | Kaburi BB, Kubio C, Kenu E, Ameme DK, Mahama JY, Sackey SO, et al. Evaluation of bacterial meningitis surveillance data of the northern region, Ghana, 2010-2015. Pan Afr Med J. 2017;27:164. Epub 2017/09/15. doi: 10.11604/pamj.2017.27.164.11036. PubMed PMID: 28904692; PubMed Central PMCID: PMCPMC5567946. |
| 63. | B | Kaburi BB, Kubio C, Kenu E, Nyarko KM, Mahama JY, Sackey SO, et al. Evaluation of the enhanced meningitis surveillance system, Yendi municipality, northern Ghana, 2010-2015. BMC Infect Dis. 2017;17(1):306. Epub 2017/04/26. doi: 10.1186/s12879-017-2410-0. PubMed PMID: 28438133; PubMed Central PMCID: PMCPMC5404286. |
| 64. | B | Kebede S, Gatabazi JB, Rugimbanya P, Mukankwiro T, Perry HN, Alemu W, et al. Strengthening systems for communicable disease surveillance: creating a laboratory network in Rwanda. Health Res Policy Syst. 2011;9:27. Epub 2011/06/28. doi: 10.1186/1478-4505-9-27. PubMed PMID: 21702948; PubMed Central PMCID: PMCPMC3142247. |
| 65. | A | Kelly-Hope LA, Blundell HJ, Macfarlane CL, Molyneux DH. Innovative Surveillance Strategies to Support the Elimination of Filariasis in Africa. Trends in parasitology. 2018;34(8):694-711. Epub 2018/07/01. doi: 10.1016/j.pt.2018.05.004. PubMed PMID: 29958813. |
| 66. | B | Khosa E, Kuonza LR, Kruger P, Maimela E. Towards the elimination of malaria in South Africa: a review of surveillance data in Mutale Municipality, Limpopo Province, 2005 to 2010. Malar J. 2013;12:7. Epub 2013/01/09. doi: 10.1186/1475-2875-12-7. PubMed PMID: 23294805; PubMed Central PMCID: PMCPMC3566928. |
| 67. | B | Kihembo C, Masiira B, Nakiire L, Katushabe E, Natseri N, Nabukenya I, et al. The design and implementation of the re-vitalised integrated disease surveillance and response (IDSR) in Uganda, 2013-2016. BMC Public Health. 2018;18(1):879. Epub 2018/07/15. doi: 10.1186/s12889-018-5755-4. PubMed PMID: 30005613; PubMed Central PMCID: PMCPMC6045850. |
| 68. | A | Kohler PK, Marumo E, Jed SL, Mema G, Galagan S, Tapia K, et al. A national evaluation using standardised patient actors to assess STI services in public sector clinical sentinel surveillance facilities in South Africa. Sex Transm Infect. 2017;93(4):247-52. Epub 2017/01/29. doi: 10.1136/sextrans-2016-052930. PubMed PMID: 28130505. |
| 69. | A | Lafond KE, Dalhatu I, Shinde V, Ekanem EE, Ahmed S, Peebles P, et al. Notifiable disease reporting among public sector physicians in Nigeria: a cross-sectional survey to evaluate possible barriers and identify best sources of information. BMC Health Serv Res. 2014;14. doi: 10.1186/s12913-014-0568-3. PubMed PMID: WOS:000348423500001. |
| 70. | A | Lim JK, Carabali M, Lee JS, Lee KS, Namkung S, Lim SK, et al. Evaluating dengue burden in Africa in passive fever surveillance and seroprevalence studies: protocol of field studies of the Dengue Vaccine Initiative. BMJ Open. 2018;8(1):e017673. Epub 2018/01/24. doi: 10.1136/bmjopen-2017-017673. PubMed PMID: 29358421; PubMed Central PMCID: PMCPMC5780679. |
| 71. | A | Lohfeld L, Kangombe-Ngwenya T, Winters AM, Chisha Z, Hamainza B, Kamuliwo M, et al. A qualitative review of implementer perceptions of the national community-level malaria surveillance system in Southern Province, Zambia. Malar J. 2016;15(1):400. Epub 2016/08/10. doi: 10.1186/s12936-016-1455-7. PubMed PMID: 27502213; PubMed Central PMCID: PMCPMC4977701. |
| 72. | A | Makoni A, Chemhuru M, Gombe N, Shambira G, Juru T, Bangure D, et al. Evaluation of the acute flaccid paralysis (AFP) surveillance system, Gokwe North district, Zimbabwe, 2015: a descriptive cross sectional study. Pan Afr Med J. 2017;27:203. Epub 2017/09/15. doi: 10.11604/pamj.2017.27.203.10956. PubMed PMID: 28904728; PubMed Central PMCID: PMCPMC5579435. |
| 73. | B | Maman I, Badziklou K, Landoh ED, Halatoko AW, Nzussouo TN, Defang GN, et al. Implementation of influenza-like illness sentinel surveillance in Togo. BMC Public Health. 2014;14:981. Epub 2014/09/23. doi: 10.1186/1471-2458-14-981. PubMed PMID: 25239536; PubMed Central PMCID: PMCPMC4190418. |
| 74. | B | Mbaeyi C, Mohamed A, Owino BO, Mengistu KF, Ehrhardt D, Elsayed EA. Strengthening Acute Flaccid Paralysis Surveillance Through the Village Polio Volunteers Program in Somalia. Clin Infect Dis. 2018;67(6):941-6. Epub 2018/03/07. doi: 10.1093/cid/ciy180. PubMed PMID: 29509835. |
| 75. | A | McLean E, Dube A, Saul J, Branson K, Luhanga M, Mwiba O, et al. Implementing electronic data capture at a well-established health and demographic surveillance site in rural northern Malawi. Global health action. 2017;10(1):1367162. Epub 2017/09/19. doi: 10.1080/16549716.2017.1367162. PubMed PMID: 28922071; PubMed Central PMCID: PMCPMC5645702. |
| 76. | A | McNamara LA, Schafer IJ, Nolen LD, Gorina Y, Redd JT, Lo T, et al. Ebola Surveillance - Guinea, Liberia, and Sierra Leone. MMWR supplements. 2016;65(3):35-43. Epub 2016/07/09. doi: 10.15585/mmwr.su6503a6. PubMed PMID: 27389614. |
| 77. | A | Merkord CL, Liu Y, Mihretie A, Gebrehiwot T, Awoke W, Bayabil E, et al. Integrating malaria surveillance with climate data for outbreak detection and forecasting: the EPIDEMIA system. Malar J. 2017;16(1):89. Epub 2017/02/25. doi: 10.1186/s12936-017-1735-x. PubMed PMID: 28231803; PubMed Central PMCID: PMCPMC5324298. |
| 78. | A | Mutonga D, Langat D, Mwangi D, Tonui J, Njeru M, Abade A, et al. National surveillance data on the epidemiology of cholera in Kenya, 1997-2010. J Infect Dis. 2013;208 Suppl 1:S55-61. Epub 2013/10/23. doi: 10.1093/infdis/jit201. PubMed PMID: 24101646. |
| 79. | A | Mutsigiri-Murewanhema F, Mafaune PT, Juru T, Gombe NT, Bangure D, Mungati M, et al. Evaluation of the maternal mortality surveillance system in Mutare district, Zimbabwe, 2014-2015: a cross sectional study. Pan Afr Med J. 2017;27:204. Epub 2017/09/15. doi: 10.11604/pamj.2017.27.204.7210. PubMed PMID: 28904729; PubMed Central PMCID: PMCPMC5579423. |
| 80. | A | Muzondo M, Shamu A, Shambira G, Gombe NT, Juru TP, Tshimanga M. Evaluation of the acute flaccid paralysis (AFP) surveillance system in Mwenezi district, Masvingo, 2018: a descriptive study. BMC Res Notes. 2018;11(1):875. Epub 2018/12/12. doi: 10.1186/s13104-018-3981-6. PubMed PMID: 30526638; PubMed Central PMCID: PMCPMC6288969. |
| 81. | B | Nadri J, Sauvageot D, Njanpop-Lafourcade BM, Baltazar CS, Banla Kere A, Bwire G, et al. Sensitivity, Specificity, and Public-Health Utility of Clinical Case Definitions Based on the Signs and Symptoms of Cholera in Africa. Am J Trop Med Hyg. 2018;98(4):1021-30. Epub 2018/03/01. doi: 10.4269/ajtmh.16-0523. PubMed PMID: 29488455; PubMed Central PMCID: PMCPMC5928804. |
| 82. | B | Ngbichi JM, Ly M, Rakotonirina J, Andrianirinarison JC, Ravaorisoa L, Harisoa J, et al. Assessing the performance of an integrated disease surveillance and response system in the context of varying malaria transmission: a case study from Madagascar. Am J Trop Med Hyg. 2017;95(5):310-. PubMed PMID: WOS:000412851502512. |
| 83. | B | Nsubuga P, Nwanyanwu O, Nkengasong JN, Mukanga D, Trostle M. Strengthening public health surveillance and response using the health systems strengthening agenda in developing countries. BMC Public Health. 2010;10 Suppl 1(Suppl 1):S5. Epub 2010/12/22. doi: 10.1186/1471-2458-10-s1-s5. PubMed PMID: 21143827; PubMed Central PMCID: PMCPMC3005577. |
| 84. | A | Nuoh RD, Nyarko KM, Nortey P, Sackey SO, Lwanga NC, Ameme DK, et al. Review of meningitis surveillance data, upper West Region, Ghana 2009-2013. Pan Afr Med J. 2016;25(Suppl 1):9. Epub 2017/02/18. doi: 10.11604/pamj.supp.2016.25.1.6180. PubMed PMID: 28210377; PubMed Central PMCID: PMCPMC5292117. |
| 85. | B | Odega CC, Fatiregun AA, Osagbemi GK. Completeness of suspected measles reporting in a southern district of Nigeria. Public Health. 2010;124(1):24-7. Epub 2010/01/13. doi: 10.1016/j.puhe.2009.11.004. PubMed PMID: 20064648. |
| 86. | B | Odoom JK, Ntim NA, Sarkodie B, Addo J, Minta-Asare K, Obodai E, et al. Evaluation of AFP surveillance indicators in polio-free Ghana, 2009-2013. BMC Public Health. 2014;14:687. Epub 2014/07/06. doi: 10.1186/1471-2458-14-687. PubMed PMID: 24996415; PubMed Central PMCID: PMCPMC4094438. |
| 87. | B | Onyebujoh PC, Thirumala AK, Ndihokubwayo JB. Integrating laboratory networks, surveillance systems and public health institutes in Africa. African journal of laboratory medicine. 2016;5(3):431. Epub 2017/09/08. doi: 10.4102/ajlm.v5i3.431. PubMed PMID: 28879136; PubMed Central PMCID: PMCPMC5433809 have inappropriately influenced them in writing this article. |
| 88. | A | Ope M, Sonoiya S, Kariuki J, Mboera LE, Gandham RN, Schneidman M, et al. Regional initiatives in support of surveillance in East Africa: The East Africa Integrated Disease Surveillance Network (EAIDSNet) Experience. Emerg Health Threats J. 2013;6. Epub 2013/01/31. doi: 10.3402/ehtj.v6i0.19948. PubMed PMID: 23362409; PubMed Central PMCID: PMCPMC3557906. |
| 89. | A | Pach A, Warren M, Chang I, Im J, Nichols C, Meyer CG, et al. A Qualitative Study Investigating Experiences, Perceptions, and Healthcare System Performance in Relation to the Surveillance of Typhoid Fever in Madagascar. Clin Infect Dis. 2016;62 Suppl 1:S69-75. Epub 2016/03/05. doi: 10.1093/cid/civ892. PubMed PMID: 26933024. |
| 90. | B | Patel P, Sabin K, Godfrey-Faussett P. Approaches to Improve the Surveillance, Monitoring, and Management of Noncommunicable Diseases in HIV-Infected Persons: Viewpoint. JMIR public health and surveillance. 2018;4(4):e10989. Epub 2018/12/24. doi: 10.2196/10989. PubMed PMID: 30573446; PubMed Central PMCID: PMCPMC6320411. |
| 91. | C | Phalkey RK, Yamamoto S, Awate P, Marx M. Challenges with the implementation of an Integrated Disease Surveillance and Response (IDSR) system: systematic review of the lessons learned. Health Policy Plan. 2015;30(1):131-43. Epub 2013/12/24. doi: 10.1093/heapol/czt097. PubMed PMID: 24362642. |
| 92. | A | Podewils LJ, Bantubani N, Bristow C, Bronner LE, Peters A, Pym A, et al. Completeness and Reliability of the Republic of South Africa National Tuberculosis (TB) Surveillance System. BMC Public Health. 2015;15:765. Epub 2015/08/12. doi: 10.1186/s12889-015-2117-3. PubMed PMID: 26259599; PubMed Central PMCID: PMCPMC4542096. |
| 93. | A | Pore M, Arnold AL, Mugambi P, Myers R, Sengeh D, Sesay T, et al. A Qualitative Evaluation of a Decision Support System for District-Level Disease Surveillance in Sierra Leone. Stud Health Technol Inform. 2018;247:451-5. Epub 2018/04/22. PubMed PMID: 29678001. |
| 94. | A | Poy A, Minkoulou E, Shaba K, Yahaya A, Gaturuku P, Dadja L, et al. Polio Eradication Initiative contribution in strengthening immunization and integrated disease surveillance data management in WHO African region, 2014. Vaccine. 2016;34(43):5181-6. Epub 2016/07/09. doi: 10.1016/j.vaccine.2016.05.057. PubMed PMID: 27389171. |
| 95. | A | Priotto G, Rguig A, Ziani M, Berger A, Nabeth P. Needs assessment for performance improvement of personnel in charge of epidemiological surveillance in Morocco. PLoS One. 2014;9(7):e101594. Epub 2014/07/08. doi: 10.1371/journal.pone.0101594. PubMed PMID: 25000499; PubMed Central PMCID: PMCPMC4084914. |
| 96. | B | Rachas A, Nakoune E, Bouscaillou J, Paireau J, Selekon B, Senekian D, et al. Timeliness of yellow fever surveillance, Central African Republic. Emerg Infect Dis. 2014;20(6):1004-8. Epub 2014/05/27. doi: 10.3201/eid2006.130671. PubMed PMID: 24857597; PubMed Central PMCID: PMCPMC4036780. |
| 97. | A | Rajput ZA, Mbugua S, Amadi D, Chepngeno V, Saleem JJ, Anokwa Y, et al. Evaluation of an Android-based mHealth system for population surveillance in developing countries. J Am Med Inform Assoc. 2012;19(4):655-9. Epub 2012/03/01. doi: 10.1136/amiajnl-2011-000476. PubMed PMID: 22366295; PubMed Central PMCID: PMCPMC3384107. |
| 98. | B | Rakotoarisoa A, Randrianasolo L, Tempia S, Guillebaud J, Razanajatovo N, Randriamampionona L, et al. Evaluation of the influenza sentinel surveillance system in Madagascar, 2009-2014. Bull World Health Organ. 2017;95(5):375-81. Epub 2017/05/10. doi: 10.2471/blt.16.171280. PubMed PMID: 28479639; PubMed Central PMCID: PMCPMC5418817. |
| 99. | A | Rasmussen SL. Practices of disease surveillance and response in Burkina Faso. In: Islam MS, Choudrie J, Wahid F, Bass JM, Priyatma JE, editors. 14th IFIP WG 94 International Conference on Social Implications of Computers in Developing Countries, ICT4D 2017: Springer New York LLC; 2017. p. 333-44. |
| 100. | B | Rha B, Dahl RM, Moyes J, Binder AM, Tempia S, Walaza S, et al. Performance of Surveillance Case Definitions in Detecting Respiratory Syncytial Virus Infection Among Young Children Hospitalized With Severe Respiratory Illness-South Africa, 2009-2014. Journal of the Pediatric Infectious Diseases Society. 2018. Epub 2018/06/23. doi: 10.1093/jpids/piy055. PubMed PMID: 29931284. |
| 101. | A | Rush T. Disease surveillance system evaluation as a model for improved integration and standardization of the laboratory component in the Field Epidemiology and Laboratory Training Program (FELTP) curriculum worldwide. J Public Health Policy. 2012;33(4):390-400. doi: 10.1057/jphp.2012.35. PubMed PMID: WOS:000310782100002. |
| 102. | C | Sahal N, Reintjes R, Aro AR. Communicable diseases surveillance lessons learned from developed and developing countries: Literature review. Scand J Soc Med. 2009;37(2):187-200. |
| 103. | A | Sanford Wesseh C, Nagbe T, Kruger J, Hamblion E, Clement P, Bawo L, et al., editors. Integrated Disease Surveillance and Response in Liberia: national expert group meeting, 15-19 September 20152016 2016/03/04/: World Health Organization. |
| 104. | B | Schoub BD. Surveillance and management of influenza on the African continent. Expert Rev Respir Med. 2010;4(2):167-9. Epub 2010/04/22. doi: 10.1586/ers.10.10. PubMed PMID: 20406082. |
| 105. | B | Sharma A, Ndisha M, Ngari F, Kipruto H, Cain KP, Sitienei J, et al. A review of data quality of an electronic tuberculosis surveillance system for case-based reporting in Kenya. Eur J Public Health. 2015;25(6):1095-7. Epub 2015/05/27. doi: 10.1093/eurpub/ckv092. PubMed PMID: 26009610; PubMed Central PMCID: PMCPMC5017003. |
| 106. | A | Sofeu CL, Broban A, Njifou Njimah A, Blaise Momo J, Sadeuh-Mba SA, Druelles S, et al. Improving systematic rabies surveillance in Cameroon: A pilot initiative and results for 2014-2016. PLoS Negl Trop Dis. 2018;12(9):e0006597. Epub 2018/09/07. doi: 10.1371/journal.pntd.0006597. PubMed PMID: 30188891; PubMed Central PMCID: PMCPMC6126802 following competing interests: AB was employed by the funder under temporary contract during the study. ML and SD are employees of the funder, Sanofi Pasteur. The other authors declare that no competing interests exist. |
| 107. | A | Somda ZC, Perry HN, Messonnier NR, Djingarey MH, Ki SO, Meltzer MI. Modeling the cost-effectiveness of the integrated disease surveillance and response (IDSR) system: meningitis in Burkina Faso. PLoS One. 2010;5(9). Epub 2010/10/12. doi: 10.1371/journal.pone.0013044. PubMed PMID: 20927386; PubMed Central PMCID: PMCPMC2946913. |
| 108. | B | Sow I, Alemu W, Nanyunja M, Duale S, Perry HN, Gaturuku P. Trained district health personnel and the performance of integrated disease surveillance in the WHO African region. East African journal of public health. 2010;7(1):16-9. Epub 2011/03/19. PubMed PMID: 21413567. |
| 109. | A | Tambo E, Ai L, Zhou X, Chen JH, Hu W, Bergquist R, et al. Surveillance-response systems: the key to elimination of tropical diseases. Infectious diseases of poverty. 2014;3:17. Epub 2014/06/28. doi: 10.1186/2049-9957-3-17. PubMed PMID: 24971165; PubMed Central PMCID: PMCPMC4071800. |
| 110. | A | Tassie JM, Bertagnolio S, Souteyrand Y. Integrated surveillance of HIV care in low-income and middle-income countries. Curr Opin HIV AIDS. 2011;6(4):233-8. doi: 10.1097/COH.0b013e328347798d. PubMed PMID: WOS:000295514200001. |
| 111. | A | Tegegne AA, Fiona B, Shebeshi ME, Hailemariam FT, Aregay AK, Beyene B, et al. Analysis of acute flaccid paralysis surveillance in Ethiopia, 2005-2015: progress and challenges. Pan Afr Med J. 2017;27(Suppl 2):10. Epub 2017/09/12. doi: 10.11604/pamj.supp.2017.27.2.10694. PubMed PMID: 28890751; PubMed Central PMCID: PMCPMC5578723. |
| 112. | A | Toda M, Zurovac D, Njeru I, Kareko D, Mwau M, Morita K. Health worker knowledge of Integrated Disease Surveillance and Response standard case definitions: a cross-sectional survey at rural health facilities in Kenya. BMC Public Health. 2018;18(1):146. Epub 2018/01/19. doi: 10.1186/s12889-018-5028-2. PubMed PMID: 29343225; PubMed Central PMCID: PMCPMC5772726. |
| 113. | A | Toyama Y, Ota M, Beyene BB. Event-based surveillance in north-western Ethiopia: experience and lessons learnt in the field. Western Pacific surveillance and response journal : WPSAR. 2015;6(3):22-7. Epub 2015/12/17. doi: 10.5365/wpsar.2015.6.2.002. PubMed PMID: 26668763; PubMed Central PMCID: PMCPMC4675161. |
| 114. | A | Umeh CA, Onyi SC. Case based rubella surveillance in Abia State, South East Nigeria, 2007-2011. PeerJ. 2014;2:e580. Epub 2014/10/08. doi: 10.7717/peerj.580. PubMed PMID: 25289179; PubMed Central PMCID: PMCPMC4183961. |
| 115. | A | Wassie E, Ademe A, Gallagher K, Braka F, Beyene B, Woyessa AB, et al. Assessment of reporting sites for acute flaccid paralysis surveillance in Ethiopia: implications for planning of active case search visits. Pan Afr Med J. 2017;27(Suppl 2):8. Epub 2017/10/07. doi: 10.11604/pamj.supp.2017.27.2.10731. PubMed PMID: 28983396; PubMed Central PMCID: PMCPMC5619921. |
| 116. | A | Weah VD, Doedeh JS, Wiah SQ, Nyema E, Lombeh S, Naiene J. Enhancing Ebola Virus Disease Surveillance and Prevention in Counties Without Confirmed Cases in Rural Liberia: Experiences from Sinoe County During the Flare-up in Monrovia, April to June, 2016. PLoS currents. 2017;9. Epub 2017/12/01. doi: 10.1371/currents.outbreaks.2b7f352af0866accbd7e5a82f165432a. PubMed PMID: 29188127; PubMed Central PMCID: PMCPMC5693337. |
| 117. | A | WHO. Integrated Disease Surveillance and Response in Liberia: national expert group meeting,15–19 September 2015. Wkly Epidemiol Rec. 2016;91(9):112-20. Epub 2016/03/08. PubMed PMID: 26946542. |
| 118. | B | Yukich JO, Butts J, Miles M, Berhane Y, Nahusenay H, Malone JL, et al. A description of malaria sentinel surveillance: a case study in Oromia Regional State, Ethiopia. Malar J. 2014;13:88. Epub 2014/03/13. doi: 10.1186/1475-2875-13-88. PubMed PMID: 24618105; PubMed Central PMCID: PMCPMC3995772. |
| 119. | A | Yusuf OB, Kabir J, Alaribe CCO. Analysis of tuberculosis surveillance data in Oyo State, Nigeria, 2011-2014. Pan Afr Med J. 2018;30(Suppl 1):1. Epub 2019/03/13. doi: 10.11604/pamj.supp.2018.30.1.15273. PubMed PMID: 30858905; PubMed Central PMCID: PMCPMC6379552. |

**A** - Study findings not based on assessment of surveillance system functions

**B** - Study findings not based on either health workers’ perspectives or health records review

**C** - Study findings based on a systematic literature reviews
