# Supplementary material for: A systematic review on improving implementation of the revitalised integrated disease surveillance and response system in the African region: A health workers’ perspective
Source: PLoS One. 2021 Mar 19;16(3):e0248998. doi: 10.1371/journal.pone.0248998 (PMC7978283; doi:10.1371/journal.pone.0248998)
Supplement: S3 Table — (DOCX) [file pone.0248998.s003.docx]

**S3 Table. Surveillance system functions (main themes and *emerging sub-themes*)**

| **CORE FUNCTIONS** | **SUPPORT FUNCTIONS** | **ATTRIBUTE FUNCTIONS** |
| --- | --- | --- |
| 1) Case detection  2) Case registration  3) Case confirmation (*Improved specimen handling; Strengthened laboratory support)*  4) Reporting *(Improved reporting quality; Adequate reporting forms provision)*  5) Data analysis *(Increased surveillance performance monitoring; Improved data accuracy)*  6) Feedback *(Improved health workers’ attitudes; Enhanced feedback from higher to lower levels)*  7) Outbreak preparedness  8) Response  9) Evaluation | 1) Standards and guidelines  2) Supervision *(Strengthened implementation of surveillance systems; Utilisation of up-to-date information; Identification of correct reporting channels)*  3) Training *(Improved performance of the surveillance system; Improved surveillance data quality; Enhanced knowledge on surveillance systems)*  4) Resources *(Financial resources; Human resources; Technical, material and logistical resources; Equipment and infrastructure)*  5) Coordination | 1) Simplicity  2) Acceptability  3) Stability  4) Flexibility  5) Usefulness  6) Data quality  7) Timeliness  8) Completeness  9) Representativeness  10) Sensitivity  11) Predictive value positive |
