# Supplementary material for: A systematic review on improving implementation of the revitalised integrated disease surveillance and response system in the African region: A health workers’ perspective
Source: PLoS One. 2021 Mar 19;16(3):e0248998. doi: 10.1371/journal.pone.0248998 (PMC7978283; doi:10.1371/journal.pone.0248998)
Supplement: S4 Table — (DOCX) [file pone.0248998.s004.docx]

**S4 Table. Summary of sub-themes with frequency and intensity effect sizes**

| Main Themes | Sub-themes | FES (%) | Nnebue et al . 2013 [27] | Abubakar et al. 2013 [32] | Maponga et al. 2014 [34] | Nnebue et al. 2014 [26] | Adokiya et al. 2015 (a) [21] | Adokiya et al. 2015 (b) [35] | Tsitsi et al. 2015 [36] | Issah et al. 2015 [29] | Lar et al. 2015 [24] | Ngwa et al. 2016 [37] | Benson et al. 2016 [20] | Mwatondo et al. 2016 [38] | Adokiya et al. 2016 [22] | Baghdadi , 2016 [39] | Adokiya and Awoonor-Wiliams, 2016 [40] | Mairosi et al. 2016 [41] | Iwu et al. 2016 [25] | Begashaw and Tesfaye, 2016 [28] | Mandyata et al. 2017 [42] | Mboera et al. 2017 [33] | Lakew et al. 2017 [43] | Haakonde et al. 2018 [23] | Dairo et al. 2018 [19] | Randriamiarana et al. 2018 [45] | Masiira et al. 2019 [46] | Ameh et al. 2016 [31] | Adjei et al. 2017 [47] | Junadu et al. 2018 [48] | Benson et al. 2017 [30] | Wu et al. 2018 [44] |
| --- | --- | --- | --- | --- | --- | --- | --- | --- | --- | --- | --- | --- | --- | --- | --- | --- | --- | --- | --- | --- | --- | --- | --- | --- | --- | --- | --- | --- | --- | --- | --- | --- |
| IES (%) | | | **27.8** | **5.6** | **5.6** | **11.1** | **11.1** | **5.6** | **22.2** | **5.6** | **11.1** | **27.8** | **5.6** | **16.7** | **5.6** | **22.2** | **5.6** | **5.6** | **11.1** | **11.1** | **11.1** | **16.7** | **22.2** | **16.7** | **5.6** | **5.6** | **16.7** | **16.7** | **5.6** | **11.1** | **0.0** | **0.0** |
| Surveillance Core  Functions | ***Case Confirmation*** |  |  |  |  |  |  |  |  |  |  |  |  |  |  |  |  |  |  |  |  |  |  |  |  |  |  |  |  |  |  |  |
|  | Provision of laboratory facilities | **6.7** |  |  |  |  |  |  |  |  |  | X |  |  |  |  | X |  |  |  |  |  |  |  |  |  |  |  |  |  |  |  |
|  | Improved specimen handling | **3.3** |  |  |  |  |  |  |  |  |  |  |  |  |  | X |  |  |  |  |  |  |  |  |  |  |  |  |  |  |  |  |
|  | ***Reporting*** |  |  |  |  |  |  |  |  |  |  |  |  |  |  |  |  |  |  |  |  |  |  |  |  |  |  |  |  |  |  |  |
|  | Provision of reporting forms | **13.3** | X |  |  |  |  |  |  |  | X |  |  |  |  | X |  |  |  |  |  |  | X |  |  |  |  |  |  |  |  |  |
|  | Reporting quality | **13.3** |  |  |  |  |  | X |  |  |  |  |  |  |  | X |  |  |  |  |  |  |  |  |  |  |  | X |  | X |  |  |
|  | ***Feedback*** |  |  |  |  |  |  |  |  |  |  |  |  |  |  |  |  |  |  |  |  |  |  |  |  |  |  |  |  |  |  |  |
|  | Improved feedback from higher to lower levels | **10.0** |  | X |  |  |  |  |  |  |  |  |  |  |  |  |  |  |  | X |  | X |  |  |  |  |  |  |  |  |  |  |
|  | Improved health workers’ attitudes | **3.3** |  |  |  |  |  |  |  |  |  |  | X |  |  |  |  |  |  |  |  |  |  |  |  |  |  |  |  |  |  |  |
|  | ***Data Analysis*** |  |  |  |  |  |  |  |  |  |  |  |  |  |  |  |  |  |  |  |  |  |  |  |  |  |  |  |  |  |  |  |
|  | Improved data accuracy and quality | **6.7** |  |  |  |  |  |  |  |  |  |  |  |  | X |  |  |  |  |  |  | X |  |  |  |  |  |  |  |  |  |  |
|  | Surveillance performance monitoring | **3.3** |  |  |  |  |  |  |  |  |  |  |  |  |  |  |  |  |  |  |  |  | X |  |  |  |  |  |  |  |  |  |
| Surveillance Support Functions | ***Training*** |  |  |  |  |  |  |  |  |  |  |  |  |  |  |  |  |  |  |  |  |  |  |  |  |  |  |  |  |  |  |  |
|  | Knowledge on surveillance systems | **33.3** | X |  |  | X |  |  | X | X |  |  |  | X |  |  |  | X |  |  |  |  |  |  |  | X | X |  | X | X |  |  |
|  | Improved surveillance data quality | **16.7** |  |  |  |  | X |  |  |  | X |  |  |  |  | X |  |  | X |  | X |  |  |  |  |  |  |  |  |  |  |  |
|  | Improved performance of the surveillance system | **10.0** |  |  | X |  |  |  |  |  |  | X |  |  |  |  |  |  |  |  |  |  |  | X |  |  |  |  |  |  |  |  |
|  | ***Supervision*** |  |  |  |  |  |  |  |  |  |  |  |  |  |  |  |  |  |  |  |  |  |  |  |  |  |  |  |  |  |  |  |
|  | Identification of correct reporting channels | **6.7** | X |  |  |  |  |  | X |  |  |  |  |  |  |  |  |  |  |  |  |  |  |  |  |  |  |  |  |  |  |  |
|  | Utilisation of up-to-date information | **3.3** |  |  |  |  |  |  |  |  |  | X |  |  |  |  |  |  |  |  |  |  |  |  |  |  |  |  |  |  |  |  |
|  | Strengthening the surveillance system | **13.3** | X |  |  |  |  |  |  |  |  |  |  |  |  |  |  |  |  |  |  |  | X | X |  |  | X |  |  |  |  |  |
|  | ***Resources*** |  |  |  |  |  |  |  |  |  |  |  |  |  |  |  |  |  |  |  |  |  |  |  |  |  |  |  |  |  |  |  |
|  | Equipment and Infrastructure | **10.0** |  |  |  |  | X |  | X |  |  | X |  |  |  |  |  |  |  |  | X |  |  |  |  |  |  |  |  |  |  |  |
|  | Technical, material and logistical resources | **20.0** |  |  |  | X |  |  | X |  |  |  |  | X |  |  |  |  |  | X |  | X |  |  | X |  |  | X |  |  |  |  |
|  | Human resources | **6.7** |  |  |  |  |  |  |  |  |  | X |  | X |  |  |  |  |  |  |  |  |  |  |  |  |  |  |  |  |  |  |
|  | Financial resources | **20.0** | X |  |  |  |  |  |  |  |  |  |  |  |  |  |  |  | X |  |  |  | X | X |  |  | X | X |  |  |  |  |

**X -** denotes presence of a given finding; **FES** **(Frequent Effect Size)** – how often a particular sub-theme appeared in the reviewed literature; **IES** **(Intensity Effect Size)** - how much each included study contributes to the reviewed literature based on number of emerging sub-themes.
