# Supplementary material for: A systematic review on improving implementation of the revitalised integrated disease surveillance and response system in the African region: A health workers’ perspective
Source: PLoS One. 2021 Mar 19;16(3):e0248998. doi: 10.1371/journal.pone.0248998 (PMC7978283; doi:10.1371/journal.pone.0248998)
Supplement: S1 File — (PDF) [file pone.0248998.s005.pdf]

NHS
National Institute for
Health Research

A systematic review of the critical findings and recommendations for the improvement of integrated disease surveillance and response systems in the African region: healthcare workers' perspectives

Arthur Kipkemoi Saitabau Ng'etich, Clifford Mutero, Kuku Voyi, Ruth Kirinyet

Citation

Arthur Kipkemoi Saitabau Ng'etich, Clifford Mutero, Kuku Voyi, Ruth Kirinyet. A systematic review of the critical findings and recommendations for the improvement of integrated disease surveillance and response systems in the African region: healthcare workers' perspectives. PROSPERO 2019 CRD42019124108 Available from:
http://www.crd.york.ac.uk/PROSPERO/display_record.php?ID=CRD42019124108

Review question

What lessons can be learned from the key findings and recommendations derived from performance assessment studies for integrated disease surveillance and response system core, support and attribute functions based on health care workers' perspectives in the African region?

Searches

We will search the following electronic bibligraphic databases: PubMed, Web of Science (Science and Social Science Citation Index), the Cumulative Index for Nursing and Allied Health Literature (CINAHL), and the World Health Organisation Library and Information Networks for Knowledge (WHOLIS).

Additionally, manual reference searches will be conducted.

Unpublished studies and relevant grey literature will also be included.

A combination of keywords and Medical Subject Headings (MeSH) relating to the integrated disease surveillance and response system will be used.

The following keywords and MeSH terms will be used in various combinations ("surveillance", "public health surveillance" [MeSH], "integrated disease surveillance and response", "integrated disease surveillance and response systems", "notifiable disease surveillance systems" "communicable disease surveillance systems" AND "surveillance core functions", "surveillance support functions", "surveillance quality attributes" AND "health workers", "health providers", "healthcare personnel" [MeSH], "health professional", "health staff" AND "Africa" [MeSH], "African region", "Sub Saharan Africa", "Africa, Eastern" [MeSH], "Africa, Western" [MeSH], "Africa, Southern" [MeSH]). A sample of the search strategy for PubMed is available in the protocol.

Restrictions: articles written in the English language only, and studies published between (01 January 2010 - 31 January 2019).

Additional search strategy information can be found in the attached PDF document (link provided below).

Search strategy

https://www.crd.york.ac.uk/PROSPEROFILES/124108_STRATEGY_20190507.pdf

Types of study to be included

We will include both quantitative and qualitative studies assessing the disease surveillance response system's core, support and attribute functions based on healthcare personnel perspectives.

Inclusion: (1) published full text articles including unpublished studies and grey literature for the period following adoption of the revised integrated disease surveillance and response (IDSR) guidelines between 2010 and 2019 by African countries; (2) either quantitative or qualitative studies or both assessing one or more functions of the IDSR systems or notifiable disease surveillance and response systems based on healthcare personnel perspectives; and (3) articles written in English language only. Other supporting studies that exclusively involve record reviews or observations to assess key functions of disease surveillance and response systems in the African region will also be included.

Exclusion: (1) articles assessing performance of disease surveillance and response systems in countries outside the African region (2) articles published prior to 2010 before the International Health Regulations (IHR 2005) was enforced in the revised IDSR guidelines adapted in the African region; (3) articles written in any other language other than English.

Condition or domain being studied

Integrated disease surveillance and response system core, support and attribute functions in the African region.

Participants/population

Healthcare workers in the African region.

Intervention(s), exposure(s)

Integrated disease surveillance and response systems in the African region: critical findings and recommendations.

The systematic review will focus on deriving critical findings and recommendations from studies assessing the performance of surveillance system core, support and attribute functions following adoption of the revised integrated disease surveillance and response (IDSR) technical guideline by a majority of African countries in 2010 and beyond. This revised guideline required strengthening of disease surveillance and response capacities through implementation of the International Health Regulations (IHR 2005) by World Health Organization (WHO) Regional Office for Africa (AFRO) member states.
The IDSR system was a strategy adopted in the African region to enable implementation of comprehensive public health surveillance and response systems. It was intended to strengthen the public health system at all levels in order to ensure prompt response to public health threats so as to alleviate illness, disability and mortality. In addition, the WHO's International Health Regulations (IHR 2005) was a legally-binding agreement providing a new framework to coordinate and manage public health threats.The IHR (2005) necessitated all WHO African member states to evaluate the ability of their national structures, capacities and resources to achieve effective surveillance and response.

Comparator(s)/control

Not applicable.

Context

The systematic review will include studies conducted in the African region. It will include African countries which adopted the revitalized integrated disease surveillance and response system technical guidelines as from 2010 and beyond.

Main outcome(s)

To systematically review the critical findings and recommendations for the improvement of integrated disease surveillance and response systems in the African region, from healthcare workers' perspectives.

This systematic review aims to inform existing health policies relating to disease surveillance and response system functions in the African region. This will be achieved through deriving the key findings and recommendations resulting from performance assessment of the integrated disease surveillance and response system core, support and attribute functions in studies previously conducted in the African region based on health care workers' perspectives.

Timing and effect measures

Not applicable.

Additional outcome(s)

None.

Timing and effect measures

Not applicable.

Data extraction (selection and coding)

All the documents and published articles will be manually reviewed, and any duplicates excluded.

The Preferred Reporting Items for Systematic Reviews and Meta-Analyses (PRISMA) Statement checklist for systematic reviews will be utilised for the review process. The PRISMA checklist has previously been used to undertake a systematic review aimed at assessing IDSR system implementation in low and middle-income countries.

The titles and abstracts of the references retrieved during the searches will be initially screened for their relevance to the main aim of the systematic review. Further to this, screening will be done to ensure that the included studies fully meet the inclusion criteria. The full texts of potentially eligible studies will be retrieved and assessed independently by each reviewer, and any discrepancies resolved between the two and, if necessary, a third reviewer will act as arbitrator.

Data will then be extracted from the studies selected for inclusion, in accordance with the PRISMA checklist, and entered into Microsoft Excel prior to analysis.

The data to be extracted will be as follows: author's name, article publication year, adoption year of IDSR revised guidelines, target disease/s, study assessment methodology, surveillance functions assessed, key findings and recommendations.

Two reviewers will independently undertake the data extraction procedure, and any discrepancies arising between the two will be resolved by discussions, and the reaching of a common consensus.

Risk of bias (quality) assessment

Dearholt and Dang's Johns Hopkins Nursing Evidence Appraisal Tool will be used for quality appraisal of the eligible studies. The quality of studies included will be based on their strength of evidence (Level I-V) and quality of evidence (Grade A, B & C). This will be done for each study included in the review by way of two authors answering a series of quality appraisal questions independently following which discrepancies will be discussed and a consensus reached on the quality of literature. The strength of evidence will be assigned level I, II, III, IV or V depending on whether the article was based on an experimental study, quasi-experimental study, non-experimental study, opinion of nationally recognized experts based on research evidence or opinion of individual expert based on non-research evidence respectively. Each eligible study will also be assigned either grade A, B or C depending on whether the quality of research evidence was of a high, good or low quality respectively. Findings from the included studies considered to have lower levels of evidence or quality in contrast to findings of higher rated studies will not be excluded from this review; however, results from these studies will be assessed more critically.

Strategy for data synthesis

Data synthesis of extracted data from quantitative studies eligible for inclusion in the review will be

accomplished by using narrative synthesis.

The analysis of the extracted data from the qualitative studies will be carried out using a thematic synthesis.

The data synthesis techniques will be structured based on pre-defined and emerging themes arising from key findings and recommendations derived from eligible studies included in the review.

Analysis of subgroups or subsets

This review will adopt a qualitative synthesis approach, and subgroup analyses may not be feasible.

Contact details for further information

Arthur Kipkemoi Saitabau Ng'etich
arthursaitabau@yahoo.com

Organisational affiliation of the review

University of Pretoria
https://www.up.ac.za/

Review team members and their organisational affiliations

Mr Arthur Kipkemoi Saitabau Ng'etich. University of Pretoria
Professor Clifford Mutero. University of Pretoria
Professor Kuku Voyi. University of Pretoria
Miss Ruth Kirinyet. Kenyatta University

Type and method of review

Epidemiologic, Narrative synthesis, Prevention, Systematic review

Anticipated or actual start date

01 April 2019

Anticipated completion date

28 June 2019

Funding sources/sponsors

None

Conflicts of interest

Language

English

Country

Kenya, South Africa

Stage of review

Review Ongoing

Subject index terms status

Subject indexing assigned by CRD

Subject index terms

Africa; Communicable Disease Control; Epidemiological Monitoring; Health Personnel; Humans; Public Health; Public Health Practice; Public Health Surveillance

Date of registration in PROSPERO

01 July 2019

Date of publication of this version

01 July 2019

Details of any existing review of the same topic by the same authors

Stage of review at time of this submission

Stage	Started	Completed
Preliminary searches	Yes	No
Piloting of the study selection process	Yes	No
Formal screening of search results against eligibility criteria	No	No
Data extraction	No	No
Risk of bias (quality) assessment	No	No
Data analysis	No	No

Versions

01 July 2019

PROSPERO
This information has been provided by the named contact for this review. CRD has accepted this information in good faith and registered the review in PROSPERO. The registrant confirms that the information supplied for this submission is accurate and complete. CRD bears no responsibility or liability for the content of this registration record, any associated files or external websites.
