# Supplementary material for: A systematic review on improving implementation of the revitalised integrated disease surveillance and response system in the African region: A health workers’ perspective
Source: PLoS One. 2021 Mar 19;16(3):e0248998. doi: 10.1371/journal.pone.0248998 (PMC7978283; doi:10.1371/journal.pone.0248998)
Supplement: S2 File — (DOCX) [file pone.0248998.s006.docx]

**S2 File. PubMed search strategy**

**MeSH and Keywords search:**

1. “Surveillance [All Fields]”

2. “Public Health Surveillance [MeSH]”

3. “Integrated disease surveillance and response”

4. “Health worker”

5. “Health personnel [MeSH]”

6. “Africa [MeSH]”

7. “Sub-Saharan Africa”

8. “Evaluation”

9. “Assessment”

10. “Publication date: 2010/01/01 – 2019/01/31”

**Combinations:**

**Group I**

((surveillance) AND africa) AND ( "2010/01/01"[PDat] : "2019/01/31"[PDat] )

((surveillance) AND health personnel) AND africa AND ( "2010/01/01"[PDat] : "2019/01/31"[PDat] )

((surveillance) AND health workers) AND africa AND ( "2010/01/01"[PDat] : "2019/01/31"[PDat] )

((surveillance) AND health personnel) AND sub-saharan africa AND ( "2010/01/01"[PDat] : "2019/01/31"[PDat] )

((surveillance) AND health workers) AND sub-saharan africa AND ( "2010/01/01"[PDat] : "2019/01/31"[PDat] )

((surveillance) AND health workers) AND africa AND evaluation AND ( "2010/01/01"[PDat] : "2019/01/31"[PDat] )

((surveillance) AND health workers) AND africa AND assessment AND ( "2010/01/01"[PDat] : "2019/01/31"[PDat] )

((surveillance) AND health personnel) AND sub-saharan africa AND evaluation AND ( "2010/01/01"[PDat] : "2019/01/31"[PDat] )

((surveillance) AND health personnel) AND sub-saharan africa AND assessment AND ( "2010/01/01"[PDat] : "2019/01/31"[PDat] )

**Group II**

((public health surveillance) AND africa) AND ( "2010/01/01"[PDat] : "2019/01/31"[PDat] )

((public health surveillance) AND health personnel) AND africa AND ( "2010/01/01"[PDat] : "2019/01/31"[PDat] )

((public health surveillance) AND health workers) AND africa AND ( "2010/01/01"[PDat] : "2019/01/31"[PDat] )

((public health surveillance) AND health personnel) AND sub-saharan africa AND ( "2010/01/01"[PDat] : "2019/01/31"[PDat] )

((public health surveillance) AND health workers) AND sub-saharan africa AND ( "2010/01/01"[PDat] : "2019/01/31"[PDat] )

**Group III**

(Integrated disease surveillance and response) AND ( "2010/01/01"[PDat] : "2019/01/31"[PDat] )

((integrated disease surveillance and response) AND africa) AND ( "2010/01/01"[PDat] : "2019/01/31"[PDat] )

((integrated disease surveillance and response) AND health personnel) AND africa AND ( "2010/01/01"[PDat] : "2019/01/31"[PDat] )

((integrated disease surveillance and response) AND health personnel) AND sub-saharan africa AND ( "2010/01/01"[PDat] : "2019/01/31"[PDat] )

((integrated disease surveillance and response) AND health workers) AND africa AND ( "2010/01/01"[PDat] : "2019/01/31"[PDat] )

((integrated disease surveillance and response) AND health workers) AND sub-saharan africa AND ( "2010/01/01"[PDat] : "2019/01/31"[PDat] )

((integrated disease surveillance and response) AND health workers) AND africa AND evaluation AND ( "2010/01/01"[PDat] : "2019/01/31"[PDat] )

((integrated disease surveillance and response) AND health workers) AND africa AND assessment AND ( "2010/01/01"[PDat] : "2019/01/31"[PDat] )

((integrated disease surveillance and response) AND health personnel) AND sub-saharan africa AND evaluation AND ( "2010/01/01"[PDat] : "2019/01/31"[PDat] )

((integrated disease surveillance and response) AND health personnel) AND sub-saharan africa AND assessment AND ( "2010/01/01"[PDat] : "2019/01/31"[PDat] )
