# Supplementary material for: A systematic review on improving implementation of the revitalised integrated disease surveillance and response system in the African region: A health workers’ perspective
Source: PLoS One. 2021 Mar 19;16(3):e0248998. doi: 10.1371/journal.pone.0248998 (PMC7978283; doi:10.1371/journal.pone.0248998)
Supplement: S3 File — (DOCX) [file pone.0248998.s007.docx]

**S3 File. Data extraction form**

**Title of the systematic review:** A systematic review on improving implementation of the revitalised integrated disease surveillance and response system in the African region: a health workers' perspective.

**Registration number:** CRD42019124108.

**1. General Information**

| **Item** | **Description** |
| --- | --- |
| 1. Date form completed  (dd/mm/yyyy) |  |
| 2. Name of person extracting data  (AKSN/RCK) |  |
| 3. Document title  (title of article/report) |  |
| 4. Publication type  (article/report/review/commentary) |  |
| 5. Country study was conducted |  |
| 6. Study ID  (i.e. surname of author and publication year) |  |
| 7. Aim of the study |  |
| 8. Disease conditions of focus |  |

**2. Eligibility**

| **Item** | **Description** |
| --- | --- |
| 9. Type of study |  |
| 10. IDSR system assessment year  (i.e. 2010 onwards when revised guidelines were adopted in Africa) |  |
| 11. Language used in the document  (i.e. English only) |  |
| 12. Decision  (i.e. with reasons for either inclusion or exclusion) |  |
| 13. Notes | |

**3. Methods**

| **Item** | **Description as stated in the document** |
| --- | --- |
| 14. Study design |  |
| 15. Study population description |  |
| 16. Sampling technique |  |
| 17. Sample size |  |
| 18. Data collection methods |  |
| Notes | |

**4. Outcomes**

| **How outcomes were measured** | **Description as stated in the document** |
| --- | --- |
| 19. Surveillance functions assessed  (core, support or attribute) |  |
| 20. Outcomes  (key findings and recommendations) |  |
| Notes: | |

**5. Results and findings**

| **Outcome 1:**  **(Note: details provided here)** | **Description as stated in the document** |
| --- | --- |
| 21. Unit of analysis |  |
| 22. Description of findings |  |
| 23. Any other results reported |  |
| Notes: | |

**6. Limitation strategy**

| **Item** | **Description as stated in the document** |
| --- | --- |
| 24. Strengths |  |
| 25. Study limitations |  |
| Notes: | |

**7. Conclusion and other information**

| **Item** | **Description as stated in the document** |
| --- | --- |
| 26. Key conclusions by study authors |  |
| Notes: |  |

**8. Risk of bias (Quality Assessment)**

| **External/Internal Validity** | **Data extractors to determine whether conditions were met (Yes/No/Not Applicable)** |
| --- | --- |
| 27. Was the study’s target population representative of health workers/personnel? |  |
| 28. Was the sample size sufficient to inform study outcomes? (based on whether it was a quantitative or qualitative study or both) |  |
| 29. Was random sampling used for quantitative studies or purposive sampling for qualitative studies? |  |
| 30. Was the likelihood of self-reporting bias minimised through other strategies? |  |
| 31. Were the data collected a reflection of the study outcomes? |  |
| 32. Were the methods for data collection and analysis appropriate? |  |
| 33. Were the conclusions and recommendations in line with the study objectives? |  |
| Notes: |  |
