# Supplementary figures and images for: A systematic review on improving implementation of the revitalised integrated disease surveillance and response system in the African region: A health workers’ perspective
Source: PLoS One. 2021 Mar 19;16(3):e0248998. doi: 10.1371/journal.pone.0248998 (PMC7978283; doi:10.1371/journal.pone.0248998)

**
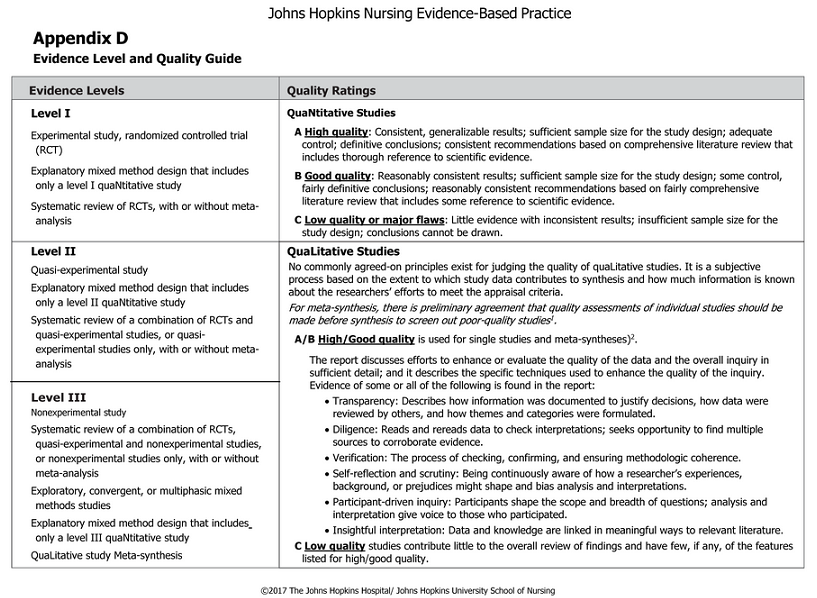
S4 File. Johns Hopkins Nursing Evidence-Based Practice Appraisal Tool**

Supplement: S4 File — (DOCX) [file pone.0248998.s008.docx]
