# Supplementary material for: A systematic review on improving implementation of the revitalised integrated disease surveillance and response system in the African region: A health workers’ perspective
Source: PLoS One. 2021 Mar 19;16(3):e0248998. doi: 10.1371/journal.pone.0248998 (PMC7978283; doi:10.1371/journal.pone.0248998)
Supplement: Attachment — Submitted filename: PONE-D-19-27712_reviewer.pdf [file pone.0248998.s009.pdf]

PLOS ONE

A systematic review on improving implementation of the revitalised integrated disease surveillance and response system in the African region: a health workers' perspective

--Manuscript Draft--

**Manuscript Number:**	PONE-D-19-27712
**Article Type:**	Research Article
**Full Title:**	A systematic review on improving implementation of the revitalised integrated disease surveillance and response system in the African region: a health workers' perspective
**Short Title:**	Improving implementation of integrated disease surveillance and response system in Africa: a systematic review
**Corresponding Author:**	Arthur Kipkemoi Saitabau Ngetich, MPH<br>University of Pretoria<br>Eldoret, KENYA
**Keywords:**	Disease surveillance and response; Health systems; Healthcare workers; Systematic review; Africa
**Abstract:**	Background: The revised integrated disease surveillance and response (IDSR) guidelines adopted by African member states in 2010 aimed at strengthening surveillance systems critical capacities. Milestones achieved through IDSR strategy implementation prior to adopting the revised guidelines are well documented; however, there is a dearth of knowledge on the progress made post-adoption. The study aimed to review key recommendations resulting from surveillance assessment studies to improve implementation of the revitalised IDSR system in the African region based on health workers' perspectives. The review focused on literature published between 2010 and 2019 post-adopting the revised IDSR guidelines in the African region.<br>Methods: A systematic literature search in PubMed, Web of Science and Cumulative Index for Nursing and Allied Health Literature was conducted. In addition, manual reference searches and grey literature searches using World Health Organisation Library and Information Networks for Knowledge databases were undertaken. The Preferred Reporting Items for Systematic Reviews and Meta-Analyses statement checklist for systematic reviews was utilised for the review process.<br>Results: Twenty-seven assessment studies met the inclusion criteria. IDSR implementation under the revised guidelines can be improved considerably bearing in mind critical findings and recommendations emanating from the reviewed surveillance assessment studies. Key recommendations alluded to provision of laboratory facilities and improved specimen handling, provision of reporting forms and improved reporting quality, surveillance data accuracy and quality, improved knowledge and surveillance system performance, utilisation of up-to-date information and surveillance system strengthening, provision of resources, enhanced reporting timeliness and completeness, adopting alternative surveillance strategies and conducting further research.<br>Conclusion: Recommendations on strengthening IDSR implementation in the African region post-adopting the revised guidelines mainly identify surveillance functions focused on reporting, feedback, training, supervision, timeliness and completeness of the surveillance system as aspects requiring policy refinement.<br>Systematic Review Registration: PROSPERO registration number CRD42019124108.
**Order of Authors:**	Arthur Kipkemoi Saitabau Ngetich, MPH
	Kuku Voyi
	Ruth Chepkosgei Kirinyet
	Clifford Maina Mutero
**Additional Information:**	
**Question**	**Response**
**Financial Disclosure**	The author(s) received no specific funding for this work.

Powered by Editorial Manager® and ProduXion Manager® from Aries Systems Corporation

Enter a financial disclosure statement that describes the sources of funding for the work included in this submission. Review the submission guidelines for detailed requirements. View published research articles from *PLOS ONE* for specific examples.<br><br>This statement is required for submission and **will appear in the published article** if the submission is accepted. Please make sure it is accurate.<br><br>**Unfunded studies**<br>Enter: *The author(s) received no specific funding for this work.*<br><br>**Funded studies**<br>Enter a statement with the following details:<br>• Initials of the authors who received each award<br>• Grant numbers awarded to each author<br>• The full name of each funder<br>• URL of each funder website<br>• Did the sponsors or funders play any role in the study design, data collection and analysis, decision to publish, or preparation of the manuscript?<br>• **NO** - Include this sentence at the end of your statement: *The funders had no role in study design, data collection and analysis, decision to publish, or preparation of the manuscript.*<br>• **YES** - Specify the role(s) played.<br><br>* typeset	
**Competing Interests**<br><br>Use the instructions below to enter a competing interest statement for this submission. On behalf of all authors, disclose any competing interests that could be perceived to bias this work—acknowledging all financial support and any other relevant financial or non-financial competing interests.<br><br>This statement **will appear in the published article** if the submission is accepted. Please make sure it is accurate. View published research articles from *PLOS ONE* for specific examples.	The authors have declared that no competing interests exist.

**NO authors have competing interests**<br><br>Enter: *The authors have declared that no competing interests exist.*<br><br>**Authors with competing interests**<br><br>Enter competing interest details beginning with this statement:<br><br>*I have read the journal's policy and the authors of this manuscript have the following competing interests: [insert competing interests here]*<br><br>* typeset	
**Ethics Statement**<br><br>Enter an ethics statement for this submission. This statement is required if the study involved:<br><br>• Human participants<br>• Human specimens or tissue<br>• Vertebrate animals or cephalopods<br>• Vertebrate embryos or tissues<br>• Field research<br><br>Write "N/A" if the submission does not require an ethics statement.<br><br>General guidance is provided below. Consult the submission guidelines for detailed instructions. **Make sure that all information entered here is included in the Methods section of the manuscript.**	N/A

Powered by Editorial Manager® and ProduXion Manager® from Aries Systems Corporation

**Format for specific study types**<br><br>**Human Subject Research (involving human participants and/or tissue)**<br>• Give the name of the institutional review board or ethics committee that approved the study<br>• Include the approval number and/or a statement indicating approval of this research<br>• Indicate the form of consent obtained (written/oral) or the reason that consent was not obtained (e.g. the data were analyzed anonymously)<br><br>**Animal Research (involving vertebrate animals, embryos or tissues)**<br>• Provide the name of the Institutional Animal Care and Use Committee (IACUC) or other relevant ethics board that reviewed the study protocol, and indicate whether they approved this research or granted a formal waiver of ethical approval<br>• Include an approval number if one was obtained<br>• If the study involved *non-human primates*, add *additional details* about animal welfare and steps taken to ameliorate suffering<br>• If anesthesia, euthanasia, or any kind of animal sacrifice is part of the study, include briefly which substances and/or methods were applied<br><br>**Field Research**<br><br>Include the following details if this study involves the collection of plant, animal, or other materials from a natural setting:<br>• Field permit number<br>• Name of the institution or relevant body that granted permission	
**Data Availability**<br><br>Authors are required to make all data underlying the findings described fully available, without restriction, and from the time of publication. PLOS allows rare exceptions to address legal and ethical concerns. See the PLOS Data Policy and FAQ for detailed information.	Yes - all data are fully available without restriction

A Data Availability Statement describing where the data can be found is required at submission. Your answers to this question constitute the Data Availability Statement and **will be published in the article**, if accepted.<br><br>**Important:** Stating 'data available on request from the author' is not sufficient. If your data are only available upon request, select 'No' for the first question and explain your exceptional situation in the text box.<br><br>Do the authors confirm that all data underlying the findings described in their manuscript are fully available without restriction?	
**Describe where the data may be found in full sentences. If you are copying our sample text, replace any instances of XXX with the appropriate details.**<br><br>• If the data are **held or will be held in a public repository**, include URLs, accession numbers or DOIs. If this information will only be available after acceptance, indicate this by ticking the box below. For example: *All XXX files are available from the XXX database (accession number(s) XXX, XXX.).*<br>• If the data are all contained **within the manuscript and/or Supporting Information files**, enter the following: *All relevant data are within the manuscript and its Supporting Information files.*<br>• If neither of these applies but you are able to provide **details of access elsewhere**, with or without limitations, please do so. For example:<br><br>*Data cannot be shared publicly because of [XXX]. Data are available from the XXX Institutional Data Access / Ethics Committee (contact via XXX) for researchers who meet the criteria for access to confidential data.*<br><br>*The data underlying the results presented in the study are available from (include the name of the third party*	All relevant data are within the manuscript and its Supporting Information files.

and contact information or URL).<br>• This text is appropriate if the data are owned by a third party and authors do not have permission to share the data.<br><br>* typeset	
Additional data availability information:	

Powered by Editorial Manager® and ProduXion Manager® from Aries Systems Corporation

Main manuscript Click here to download Manuscript Manuscript_PLOS One.docx

A systematic review on improving implementation of the revitalised integrated disease surveillance and response system in the African region: a health workers' perspective

Arthur K. S. Ng'etich[1*], Kuku Voyi[1], Ruth C. Kirinyet[2], Clifford M. Mutero[1,3,4]

[1]School of Health Systems and Public Health (SHSPH), University of Pretoria, South Africa

[2]School of Public Health, Department of Environmental and Occupational Health, Kenyatta University, Nairobi, Kenya

[3]University of Pretoria Institute for Sustainable Malaria Control (UP ISMC), University of Pretoria, South Africa.

[4]International Centre of Insect Physiology and Ecology, Nairobi, Kenya.

*Corresponding author - Arthur K. S. Ng'etich; arthursaitabau@yahoo.com (AKSN)

Abstract

Background: The revised integrated disease surveillance and response (IDSR) guidelines adopted by African member states in 2010 aimed at strengthening surveillance systems critical capacities. Milestones achieved through IDSR strategy implementation prior to adopting the revised guidelines are well documented; however, there is a dearth of knowledge on the progress made post-adoption. The study aimed to review key recommendations resulting from surveillance assessment studies to improve implementation of the revitalised IDSR system in the African region based on health workers' perspectives. The review focused on literature published between 2010 and 2019 post-adopting the revised IDSR guidelines in the African region.

Methods: A systematic literature search in PubMed, Web of Science and Cumulative Index for Nursing and Allied Health Literature was conducted. In addition, manual reference searches and grey literature searches using World Health Organisation Library and Information Networks for Knowledge databases were undertaken. The Preferred Reporting Items for Systematic Reviews and Meta-Analyses statement checklist for systematic reviews was utilised for the review process.

Results: Twenty-seven assessment studies met the inclusion criteria. IDSR implementation under the revised guidelines can be improved considerably bearing in mind critical findings and recommendations emanating from the reviewed surveillance assessment studies. Key recommendations alluded to provision of laboratory facilities and improved specimen handling, provision of reporting forms and improved reporting quality, surveillance data accuracy and quality, improved knowledge and surveillance system performance, utilisation of up-to-date information and surveillance system strengthening, provision of resources, enhanced reporting timeliness and completeness, adopting alternative surveillance strategies and conducting further research.

Conclusion: Recommendations on strengthening IDSR implementation in the African region post-adopting the revised guidelines mainly identify surveillance functions focused on reporting, feedback, training, supervision, timeliness and completeness of the surveillance system as aspects requiring policy refinement.

Systematic Review Registration: PROSPERO registration number CRD42019124108.

Introduction

Public health surveillance involves continuous collection, analysis and interpretation of health data resulting in timely information dissemination enabling effective public health action [1]. Public health surveillance systems form a critical part of information systems as a key component within the World Health Organization (WHO) health system framework [2]. The International Health Regulations (IHR 2005) within the health system are a legally binding agreement providing a framework to coordinate and manage public health threats [3, 4]. The IHR (2005) necessitated all WHO member states to evaluate ability of their national structures, capacities and resources to achieve effective disease surveillance and response [3]. Prior to IHR (2005), the WHO Regional Office for Africa (WHO-AFRO) and its member states adopted the Integrated Disease Surveillance and Response (IDSR) system [5]. IDSR system framework provided a platform to improve national public health surveillance and response capacities. The IDSR system aims to strengthen the public health system at community, health facility (HF), district, and national levels to ensure timely detection, confirmation and response to public health threats to alleviate illness, disability and mortality [5, 6]. IDSR and IHR frameworks share a common goal of improving timely response to public health events through early detection, notification, verification, response and collaboration activities [3, 6]. Therefore, member states in WHO African region declared IHR

(2005) implementation was to be achieved within the existing IDSR strategy [6]. A review of IDSR guidelines in 2010 was necessary to meet the requirements of disease surveillance and response core capacities strengthening as specified through IHR (2005) implementation by African member states [5-7].

The revised IDSR guidelines considered the recommended tools and approaches from IHR (2005) to supplement early warning capabilities in the national surveillance systems and tackle other threats to public health [6]. By 2016, 42 out of 47 countries in the African region had adopted the second edition of the revitalized IDSR technical guidelines [8]. Even though IDSR system adoption by African countries was the most pragmatic approach given resource constraints, there is paucity of knowledge as to the vital recommendations emanating from assessing IDSR system functions [3]. Hence, this review focused on surveillance assessment studies undertaken post-IHR (2005) adoption, which is in line with implementation of the revitalised IDSR system in Africa.

The health workforce across all health system levels are instrumental to achieving effective IDSR system implementation. Hence, giving due consideration to health personnel involvement and their perspectives on full optimisation of surveillance and response systems functionalities is vital to surveillance system improvement. There is insufficient review of literature on evaluation of key policy priorities based on health workers' perspectives ensuing from previous IDSR system assessment studies, which are pertinent to achieving communicable disease control in Africa. Previous systematic reviews have a limited focus on critically assessing fundamental recommendations arising from assessment studies regarding IDSR system improvement since adopting the revised IDSR guidelines in Africa [9, 10]. Identifying recommendations derived from

studies assessing the performance of IDSR system functions is key to focusing decision makers on the critical policy priority areas and guiding implementers towards improving disease surveillance and strengthening the overall health system. Therefore, the current review aimed to derive key recommendations resulting from IDSR system core, support and attribute functions assessment studies to improve implementation of the revitalised IDSR system in the African region based health workers' perspectives.

Research Question

What lessons can be learned from recommendations derived from previous IDSR system core, support and attribute functions assessment studies to improve implementation of the revitalised IDSR system in the African region based on health workers' perspectives?

The PICO (Population/Interest/Context/Outcome) framework [11, 12] modified to suit qualitative review questions was used to identify keywords in the research question (Table 1).

Table 1. PICO framework summary

Population	Phenomenon of Interest	Context	Outcome
"health workers" OR "health providers" OR "healthcare personnel" OR "health professional" OR "health staff"	"surveillance" OR "public health surveillance" OR "integrated disease surveillance and response systems" AND "surveillance core functions" OR "surveillance support functions" OR "surveillance quality attributes"	"Africa" OR "African region" OR "Sub Saharan Africa"	Key recommendations for improving the surveillance functions based on previous assessment studies for IDSR system

Methods

A protocol for this systematic review was registered on July 1, 2019 in the International Prospective Register of Systematic Reviews (PROSPERO), registration number CRD42019124108. The review focused on literature published between 2010 and 2019 post-revised IDSR guidelines adoption in Africa. The search included published articles and grey literature for the period between 1st January 2010 to 31st January 2019. Systematic literature searches in PubMed, Web of Science and Cumulative Index for Nursing and Allied Health Literature (CINAHL) using keywords search strategy, in addition to manual reference searches were undertaken. Grey literature searched using World Health Organisation Library and Information Networks for Knowledge (WHOLIS). Each database was searched in consultation with the information specialist of the University of Pretoria Health Faculty Library. Keywords combination using Medical Subject Heading (MeSH) and free text terms relating to the IDSR system were used. The following keywords were used in various combinations ("surveillance", "public health surveillance" [MeSH], "integrated disease surveillance and response", "integrated disease surveillance and response systems", AND "surveillance core functions", "surveillance support functions", "surveillance quality attributes" AND "health workers", "health providers", "healthcare personnel" [MeSH], "health professional", "health staff" AND "Africa" [MeSH], "African region", "Sub Saharan Africa". Individual search terms were combined using the appropriate Boolean operators to generate a list of citations that were saved into Endnote X8 and screened for duplicates.

The review focused on deriving key recommendations based on IDSR system's core and support functions as has been defined by WHO as well as surveillance systems attributes as defined by

Centers for Disease Control and Prevention (CDC) [13, 14]. The inclusion criteria required the literature reviewed be; (1) published full text articles including unpublished studies and grey literature for the period between 2010 and 2019; (2) either quantitative or qualitative studies or both assessing one or more IDSR system functions or notifiable disease surveillance and response systems based on health personnel (HP) perspectives; and (3) articles written in English language only. In addition, studies involving record reviews or observations to assess disease surveillance and response systems functions in the Africa were included. Exclusion criteria considered articles on surveillance assessment studies in countries outside Africa, articles published prior to 2010 before countries adopted the revised IDSR guidelines and articles written in any other language other than English.

Data extraction and synthesis

All documents and published articles were manually reviewed with duplicates excluded. Preferred Reporting Items for Systematic Reviews and Meta-Analyses (PRISMA) Statement checklist for systematic reviews was utilised in the review process [15]. Data extracted using PRISMA checklist was entered into Microsoft Excel prior to analysis. Two reviewers (AKSN, RCK) undertook data extraction and discrepancies between the two resolved by consensus. Data synthesis for quantitative studies was conducted narratively [16]. Analysis of extracted data from qualitative studies was done using thematic synthesis [17]. Both data synthesis techniques were used to identify the key themes arising from recommendations derived from studies included in the review.

Literature quality appraisal

Dearholt and Dang’s Johns Hopkins Nursing Evidence Appraisal Tool was used for quality appraisal of the reviewed literature. Quality of studies included was based on their strength of evidence (Level I-V) and quality of evidence (Grade A, B & C) [18]. This was done for each article

included in the review by two authors (AKSN, RCK) answering a series of quality appraisal questions independently following which differences were discussed and a consensus reached on quality of literature to be included in the review. The strength of evidence was assigned level I, II, III, IV or V depending on whether the article was based on an experimental study, quasi-experimental study, non-experimental study, nationally recognized experts' opinion based on research evidence or individual expert opinion based on non-research evidence respectively. Furthermore, each included article was assigned grade A, B or C depending on whether the quality of research evidence was of a high, good or low quality respectively [18]. Findings from articles considered to have lower levels of evidence or quality in contrast to findings of higher rated articles were not excluded from this review. However, results from these articles were assessed more critically.

Risk of bias across studies

Majority of included studies except those supported by document reviews and observations depended on self-reporting by healthcare workers (HCWs). This may have biased their responses towards what they felt was socially desirable at the time of conducting the studies. Secondly, the review focused on assessment studies conducted in the African region, which may have limited the study's perspective from drawing lessons based on IDSR implementation outside Africa. The review only included studies written in English language, which may have introduced some form of selection bias. Lastly, the review was based on extracting relevant studies from four databases; hence limiting the search to what was available in these databases only.

Results

Summary of included studies

The systematic search identified cumulatively 7,415 records from all the databases including a manual reference search. Records retrieved included; 6,203 articles in PubMed, 1,069 articles in Web of Science, 112 articles in CINAHL, 18 grey literature records in WHOLIS and 13 manually searched references as described in the PRISMA flow diagram (Fig 1).

Fig 1. Flow chart summarising the systematic review process

Abstracts of identified studies were reviewed and the full body text of selected articles read. All identified articles were written in English language. Of the 27 studies meeting the inclusion criteria, 23 were assessment studies exclusively based on health workers' perspectives while three of the included studies combined both health workers' views and records review, with only one study exclusively based on records review. Surveillance assessment studies were based in 13 countries in WHO-AFRO region (Ghana, Cameroon, Nigeria, Kenya, Ethiopia, Tanzania, Zimbabwe, Zambia, South Africa, Madagascar, Uganda, South Sudan and Malawi). These countries adopted the revitalised IDSR guidelines between 2010 and 2016 [8].

The included assessment studies were based on the revised African IDSR technical guideline disease categories, with twenty studies focused on notifiable diseases requiring immediate reporting while three [19-21] out of the twenty studies mentioned diseases targeted for elimination and eradication including neglected tropical diseases such as guinea worm disease, trachoma and schistosomiasis. However, seven studies did not specify any particular disease in the assessment [22-28]. The reviewed studies covered a combination of surveillance functions with 21 focusing on core functions, 20 on support functions and 15 on surveillance attribute functions. Table 2 gives

a summary of specific components covered under each surveillance function. In addition, Table 2 summarises findings from the included assessment studies. Table 3 gives an overview of recommendations to improve IDSR system implementation derived from the included studies categorised based on surveillance functions. Findings were summarised based on specific surveillance functions highlighting recommendations derived from the reviewed studies.

According to Dearholt and Dang's Johns Hopkins Nursing Evidence Appraisal Tool [18], most studies were assigned level III in terms of evidence strength since 25 out of 27 (93%) of the studies adopted a cross-sectional study design (Table 2). In addition, three [28-30] studies were considered of low quality (Grade C) in terms of evidence quality considering their methodological approach. However, since these studies satisfied the inclusion criteria, they were included in the review and their study findings critically reviewed.

Table 2. Literature summary and quality appraisal

No.	Country	Year country adopted revised IDSR guideline	Case disease/s mentioned in the assessment study	Authors	Publication Year	Aim of the study	Assessment methodology	Surveillance system functions assessed	Key Findings	Evidence Levels[(a)] and Quality Ratings[(b)]
**1.**	Nigeria	2013	Not specified	Nnebue et al.[27]	2013	To determine the completeness and timeliness of data collection and disease notification<br><br>To ascertain the pattern of transmission of information from the health facility level to the state level<br><br>To come up with ways in which the system can be improved	-A cross-sectional study<br>-Included healthcare workers involved in disease surveillance and notification in health facilities and the state epidemiologist<br>-Multistage sampling technique was used to select 5 healthcare providers from 9 health facilities from each of the 6 local government areas<br>-Sample size of 270 healthcare workers<br>-Data collection was done using interviewer‐administered healthcare provider questionnaires, health facility observational checklist, desk review, and key informant interviews	Core functions: Case registration, reporting, feedback<br><br>Attributes: Data accuracy, Reporting completeness and timeliness	-Health facilities had an inadequate supply of IDSR reporting forms<br>-Surveillance data reported through the disease surveillance and notification system were not complete and timely	III, B

**2.**	Nigeria	2013	Diarrhoea, Measles	Abubakar et al.[29]	2013	To assess the implementation of integrated disease surveillance and response in selected Local Government Areas in Kaduna State.	-Cross-sectional descriptive study<br>-Multistage sampling was used to select seven random health facilities in each of the three Local Government Areas selected from three senatorial zones<br>-Target population included Medical Officers, Disease Surveillance and Notification Officers and the State Epidemiologist<br>-Records and reports were reviewed at the health facilities, local government area health offices and state epidemiology unit<br>-Interviewer administered questionnaire was used for data collection	Core functions: Reporting, Feedback, Data analysis<br><br>Support functions: Standards and guidelines, Resources	-Poor IDSR implementation<br>-Insufficient surveillance resources<br>-Poor feedback from the higher to lower levels<br>-Poor utilisation of standard case definitions in health facilities	III, C
**3.**	Zimbabwe	2012	Cholera, Anthrax, Rabies	Maponga et al.[31]	2014	To evaluate the notifiable disease surveillance system (NDSS) in Sanyati district	-A descriptive cross sectional study conducted in selected health facilities (private and public & urban and rural)<br>-The study population included nurses, environmental health personnel, and doctors in health facilities.	Attributes: Acceptability, flexibility, simplicity, stability, data quality, timeliness, sensitivity, representativeness	-Notifiable disease surveillance system was useful, acceptable, simple, sensitive and timeliness was good<br>-The system was threatened by lack of reporting forms, poor quality of surveillance data, low feedback and lack of knowledge amongst health workers on the system	III, B

							-Key informants included health managers from rural and urban health sectors<br>-66 health workers		-The time and cost of transporting reporting forms to the next level was unsustainable	
**4.**	Nigeria	2013	Not specified	Nnebue et al.[26]	2014	To determine the functional status of the disease surveillance and notification system and examine challenges of data collection and disease notification across all surveillance levels	-A cross-sectional descriptive study<br>-Included healthcare workers involved in disease surveillance and notification in health facilities including doctors, nurses, community health extension workers and community health officers<br>-9 health facilities were selected from each of these 6 local government areas and 5 healthcare providers were selected from each of the health facilities,<br>-Sample size: 270 healthcare workers<br>-Data collection was done using interviewer administered semi-structured healthcare provider questionnaires, health facility observation checklist and key informant interviews	Core functions: Reporting, Feedback<br><br>Support functions: Supervision, Training, Resources	-Challenges associated with disease surveillance and notification included; lack of training, lack of transportation, poor motivation, inadequate supply of forms and other logistics, poor funding, ignorance on the part of the public, inadequate supervision, and lack of prompt feedback	III, A

**5.**	Ghana	2011	Malaria, HIV/AIDS, Cholera, Tuberculosis, Pneumonia, Meningitis, Poliomyelitis, Guinea Worm Disease	Adokiya et al.[21] (a)	2015	To evaluate the IDSR functioning and its data quality on the district health information management system (DHIMS)in northern Ghana	-Observational study using mixed methods (convergent parallel design)<br>-Quantitative-weekly/monthly surveillance reports<br>-Qualitative-interviews with 6 key informants by use of semi-structured questionnaires<br>-Purposive sampling of Upper East Region of Ghana given remoteness therefore higher likelihood of disease epidemics and surveillance problems	Core functions: Case detection, confirmation, reporting, data analysis, epidemic preparedness and feedback.<br><br>Support functions: Supervision, training and resources	-There are major challenges to system functioning including validity and quality of data, confirmation practices of specific diseases, problems with supervision and support at the periphery level, ill-equipped laboratories, missing feedback and low priority for surveillance<br><br>Study limitations: conducted in a limited geographical area and the study districts were not randomly selected. Therefore, study findings are not representative of the whole of Ghana	III, A
**6.**	Ghana	2011	HIV/AIDS, Tuberculosis,	Adokiya et al.[32] (b)	2015	To assess the core and support functions of the IDSR system at the periphery level of the health systems in northern Ghana	-Assessment method: A qualitative study conducted among 18 key informants responsible for health care delivery at the periphery of the health system<br>-Sampling: Convenience sampling of one region in the northern part of Ghana	Core functions: Case detection, registration, confirmation, data reporting, data analysis, epidemic response and feedback.<br><br>Support functions: Supervision, training and resources	-IDSR strategy contributed to increased surveillance report submission<br>-IDSR enhanced surveillance data analysis at the periphery health facilities<br>-IDSR improved human resource by having a designated disease surveillance officer in each health centre<br>-Supervision of surveillance activities at the peripheral level was inadequate<br>-Inadequate training for effective and efficient disease surveillance<br>-Challenges of inadequate staff, inadequate funding,	III, B

									frequent staff turnover and poorly equipped laboratories<br><br>Study Limitations:<br>-Limited to a small area in North of Ghana<br>-Most of the information was self-reported and thus influenced by individual factors of the few respondents	
**7.**	Zimbabwe	2012	Malaria, Rabies, Polio, Measles, Tuberculosis	Tsitsi et al.[33]	2015	To evaluate the notifiable disease surveillance system (NDSS) in Beitbridge district	-A descriptive cross sectional study conducted in selected health facilities (both urban & rural facilities)<br>-Study area: Beitbridge district was purposively sampled because it has a referral facility receiving a high volume of patients<br>-Study population: Nurses, environmental health technicians, health information officers and doctors<br>Key informants: 4 (health managers)<br>-53 health workers included in the study	Attributes: Acceptability, Usefulness, flexibility, simplicity, stability, sensitivity, data quality, representativeness and timeliness	-The surveillance system was acceptable, flexible and simple<br>-The system was unstable, not sensitive and not useful<br>-Reasons for under-performance of the surveillance system were lack of reporting forms, reporting guidelines and knowledge of health workers on the surveillance system<br>-Costs of reporting a single case of disease was unreasonably high using the paper-based system	III, B
**8.**	Ghana	2011	Ebola	Issah et al.[34]	2015	To assess the usefulness of integrated disease surveillance and response on	-Longitudinal study design<br>-Assessed core, support and quality functions of the surveillance system relating to ebola viral	Core functions: Case detection, Case registration, Case confirmation, Reporting, Epidemic preparedness and response.	-Over-dependence by health staff on hemorrhage as the only symptom of EVD and non-utilisation of case definitions	II, B

						suspected Ebola cases	disease using in-depth interviews with 7 medical directors and 2 district directors in addition to document reviews.	Support functions: Standards and guidelines, Training, Communication, Coordination, Resources, Monitoring and evaluation.<br><br>Attributes: Reporting timeliness	-Poor registration of suspected EVD cases robbing the system of vital information for effective decision-making<br>-Good laboratory support in terms of timely collection, transport, testing of blood samples and prompt receipt of test results.<br>-Inadequate training of health staff and community based surveillance volunteers on EVD epidemic preparedness<br>- Poor practices of blood sample collection in the community without privacy resulting in stigmatization	
**9.**	Nigeria	2013	Not specified	Lar et al.[24]	2015	Challenges of IDSR reporting among healthcare personnel in Mangu, Plateau State, Nigeria	-Quasi-experimental study<br>-Sample: 108 health personnel of primary health care facilities, 84 from public facilities and 24 from private facilities, selected using random sampling<br>-Structured, interviewer-administered questionnaires and observation checklists were used for data collection	Core functions: Reporting<br><br>Support functions: Training	-Mean level knowledge of the respondents increased post-training<br>-There was a statistically significant association between training and reporting form availability, recognition of form filling efforts and feedback	II, A

**10.**	Cameroon	2011	Cholera	Ngwa et al.[35]	2016	To assess the IDSR strategy activities in Cameroon with a particular focus on cholera; opportunities and challenges	-Use of semi-structured key informant interviews, field visits coupled with review of literature and government documents<br>-Thirty health personnel were involved in the study	Core functions: Case detection, Reporting, Outbreak detection and Feedback<br><br>Support functions: Standard guidelines, Training, Supervision, Resources and Laboratory capacity<br><br>Attributes: Completeness and timeliness of reporting	-Lack of mobile phones in the health facilities might delay immediate reporting of suspected cases of cholera<br>-Lack of data analysis and interpretation at the health facility and district levels<br>-Use of outdated cholera case definitions<br>-Health facilities completely lack laboratories or have ill-equipped laboratories<br><br>Study limitations:<br>-Selection bias of focal persons from only two regions with frequent cholera epidemics out of the ten regions	III, B
**11.**	South Africa	2013	33 notifiable conditions in South Africa (among them are diseases targeted for elimination such as Trachoma)	Benson et al.[20]	2016	To determine the perceptions of key stakeholders on the national notifiable disease surveillance system attributes	-A cross-sectional survey of key stakeholders (i.e. communicable disease coordinators and surveillance officers and members of NDSS committees) using an electronic semi-administered questionnaire	Attributes: Acceptability, Flexibility, Simplicity, Timeliness and Usefulness	-The majority of key stakeholders scored the NDSS as low on the attributes of acceptability, flexibility and usefulness.<br>-Factors associated with key stakeholders perceptions were years of experience, NDSS training, age below 35 years, participation in disease detection and national outbreak response team	III, B
**12.**	Kenya	2012	35 priority diseases as provided in the IDSR technical guideline	Mwatondo et al.[36]	2016	To determine the prevalence of adequate reporting	-Cross-sectional survey of health facilities in Nairobi County<br>-Sampling: Stratified random sampling	Core functions: Reporting<br><br>Support functions: Standards and guidelines (i.e. case	-Sub-optimal reporting of IDSR priority diseases in a majority of health facilities<br>-Health facilities submitted complete and timely reports	III, A

						and factors associated with IDSR reporting among health facilities	used to select the health facilities (i.e. public and private)<br>-Sample size: 183 health facilities<br>-Structured questionnaire administered to the health worker responsible for surveillance or officer-in-charge of reporting activities in each health facility<br>-Data collection tool was adapted from WHO protocol for assessment of national communicable disease surveillance and response and Kenya IDSR technical guidelines	definitions), resources (i.e. computer hardware and internet)<br><br>Attributes: Timeliness and completeness of surveillance reports	-Availability of reporting forms, having a surveillance focal person and having posters displaying IDSR functions were associated with adequate reporting<br><br>Study Limitations:<br>-Timeliness of reports was calculated based on the date stated on the reporting form which might have not reflected the true date the report was submitted<br>-The study was conducted in only one county in Kenya and not generalizable to the whole country	
**13.**	Ghana	2011	Not specified	Adokiya et al.[22]	2016	To evaluate the reporting completeness and timeliness of the IDSR system at the sub-national level in northern Ghana	-Observational study: A quantitative study design<br>-Study setting: Upper East region of Ghana in two districts and their nine health facilities purposively chosen because of their remoteness	Attributes: Reporting completeness and timeliness, data accuracy	Implementation of DHIMS2 showed improvements in IDSR data weekly/monthly reporting (i.e. timeliness and completeness) at the sub-national level<br><br>-Study limitations:<br>Study was conducted in a limited area of the country and based on a short duration of DHIMS2 implementation	III, B
**14.**	Sudan	2013	Meningitis	Baghdadi[37]	2016	To assess the core and support functions of	-Interview questionnaire and checklist were used to collect data from	Core functions: Case registration and confirmation, Reporting, Feedback	-Case confirmation was weak and absent in rural hospitals	III, B

						the surveillance system with regards to meningitis	31 government hospitals<br>-Questionnaire was adapted from the WHO generic questionnaire	Support functions: Standards and guidelines (case definitions), Training, Laboratory capacity, Communication facilities,	-52% of the hospitals had health personnel trained on surveillance<br>-42% of the hospitals received feedback reports from their localities<br><br>Study limitations<br>-Limited to hospitals only<br>-Not all hospitals were visited physically<br>-Dependency on telephone interviews<br>-Not all core and support functions were assessed	
**15.**	Ghana	2011	Ebola	Adokiya and Awoonor-Williams[38]	2016	To assess the Ebola Virus Disease surveillance and response system in northern Ghana	-Observational cross-sectional study<br>-Sample: 47 health care workers in 13 districts<br>-Target population: Directors of district health services, medical doctors, surveillance officers, laboratory officers of public, private and mission hospitals<br>-Interviews conducted using semi-structured questionnaires regarding core and support functions of the surveillance system and satisfaction amongst health workers of the Ebola surveillance system	Core functions: Case detection and confirmation, reporting, feedback, outbreak preparedness<br><br>Support functions: supervision, training, resources	-Lack of registers for Ebola data<br>-Inadequate Personal Protective Equipment (PPEs)<br>-Inadequate laboratory capacity<br>-Delays in specimen taking	III, B

**16.**	Zimbabwe	2012	Typhoid	Mairosi et al.[39]	2016	To evaluate the notifiable disease surveillance system (NDSS) in Centenary, Zimbabwe by assessing the usefulness of the NDSS, the systems attributes, health care worker knowledge levels and cost of running the system	-Descriptive cross sectional study design used to evaluate the NDSS using the updated CDC Guidelines for Surveillance System Evaluation.<br>-Health personnel involved in the NDSS including nurses, doctors, environmental health practitioners and health information officers were purposively selected and interviewed.<br>-Interviewer-administered questionnaire was used to interview the health workers<br>-Thirteen health facilities were included<br>-Records review of T1 forms was undertaken.<br>-Sample size: 59 health workers and 16 T1 forms	Core functions: Reporting<br><br>Attributes: Usefulness, simplicity, acceptability, stability, flexibility sensitivity, data quality and timeliness	-Low levels of knowledge on notifiable disease surveillance system resulting to missing notifiable diseases, underreporting and poor case management<br>-The notifiable disease surveillance system was unstable and lacked sensitivity	III, B
**17.**	Nigeria	2013	Not specified	Iwu et al.[25]	2016	To identify gaps in disease reporting among health care workers within a resource limited	-Cross sectional descriptive design<br>-Stratified simple random sampling technique used to select 449 health care workers<br>-Study population comprised of health care workers	Core functions: Reporting<br><br>Support functions: Training, Resources	-Most respondents perceived inadequate training, lack of equipment and inadequate supply of reporting forms as limiting factors to disease reporting	III, A

						setting in order to develop programs that improve their knowledge, attitude and practices	including physicians, nurses, community health extension workers, medical record officers and laboratory technicians<br>-Semi structured self-administered questionnaires with an observational checklist used for data collection			
**18.**	Ethiopia	2010	Not specified	Begashaw and Tesfaye[28]	2016	To assess the implementation of integrated disease surveillance and response in selected health facilities in Dawuro Zone	-Cross sectional facility based descriptive study<br>-Multi stage sampling used to select three random health facilities in each of the three randomly selected districts out of six districts in a zone<br>-The target population included; IDSR focal persons and health facility in-charges<br>-Data was collected using an interviewer administered questionnaire	Core functions: Reporting, Feedback, Data analysis,<br><br>Support functions: Resources	-Majority of health facilities had no form of data analysis available<br>-The available surveillance resources such as bicycles, motorcycles, computers and printers were non-functional in most health facilities<br>-Feedback reports from the regional level to the district level and from the zone to the health centers were unavailable	III, C
**19.**	South Africa	2013	Measles, Meningoccal Meningitis, Typhoid	Benson et al.[40]	2017	To compare laboratory surveillance with the notifiable diseases surveillance	-A retrospective records review using data from the National Health Laboratory Services and records of notifications held at	Attributes: data quality, stability, representativeness, sensitivity and positive predictive value	-Laboratory data completeness was higher for measles and meningitis but not for typhoid<br>-Both stability and representativeness was higher for laboratory	III, A

						system (NDSS)	the National Department of Health		compared to notified measles, meningitis and typhoid	
**20.**	Zambia	2012	Dysentery, Malaria, HIV, Tuberculosis, Typhoid, Measles	Mandyata et al.[41]	2017	To investigate and report on the existing challenges in the implementation of the IDSR strategy in a resource limited country from a health worker perspective	-Qualitative study approach<br>-Thirteen key informant interviews with purposively sampled health workers at all levels of IDSR implementation using interview guides<br>-Key informant interview guides were adapted from the WHO protocol for the assessment of national communicable disease surveillance and response systems and the communicable disease surveillance and response systems: guide to monitoring and evaluating	Core functions: Case detection, confirmation, registration, reporting, data analysis, response and control, feedback.<br><br>Support functions: Training, logistical support, supervision.<br><br>Attributes: Representativeness, stability	-Major successes included operationalised epidemic preparedness and response plans, full –time staff, dedicated disease surveillance budgets and adoption of IDSR technical guidelines across all levels<br>-Major challenges included inadequate trained human resource, poor infrastructure and coordination challenges.<br><br>Study limitation:<br>-The study findings from two conveniently sampled districts were perceived to be transferable to other districts throughout the country.<br>-Only certain key areas from each of the four components of the IDSR strategy were selected to highlight some of the main challenges	III, B
**21.**	Tanzania	2011	Malaria	Mboera et al.[30]	2017	To assess malaria surveillance system at the facility and district levels in order to identify key barriers, constraints and priority	-Cross-sectional study design using qualitative methods(i.e. in-depth interviews, observation and documentary review)<br>-Involved 20 health facility workers and members of the district health	Core functions: Case registration, Reporting, Data analysis, Response, Feedback,<br><br>Support functions: Standards and guidelines, Training, Resources, Communication,	-Poor data management, inefficient reporting, shortage of data collection and processing tools, limited data analysis capacity, over-burdened health staff, weak communication systems, weak capacity for facility level decision making, multiple surveillance systems demanded by	III, C

						actions for malaria surveillance strengthening<br><br>To explore the use of evidence in health planning and decision making at the facility and district levels	management team recruited purposively<br>-Evaluation of surveillance system on malaria was done using a structured questionnaire and observation checklist	Attributes: Timeliness, Completeness, Usefulness,	different vertical programmes	
**22.**	Ethiopia	2010	Vaccine Preventable Diseases i.e. Acute Flaccid Paralysis, Measles and Neonatal Tetanus	Lakew et al.[42]	2017	To assess the performances of disease surveillance and routine immunization with particular focus on vaccine preventable diseases (VPD) in Amhara Region, Ethiopia	-A cross-sectional study<br>-Purposive sampling of five zones and two town administrations out of 10 zones and 3 town administrations respectively.<br>-Primary data collection tool was a structured questionnaire adapted from WHO-AFRO to external surveillance review.<br>-The tool was administered through interviews of surveillance focal persons or their subordinates, heads of health offices, health facilities and holy water site.<br>-Document reviews (i.e. meeting minutes,	Core functions: Reporting, monitoring and evaluation<br><br>Support functions: Supervision, training, surveillance guidelines and case definitions, laboratory capacity, coordination, meetings	-Less than half of the health offices and health facilities had clearly written and communicated terms of reference and standard operating procedures for their surveillance focal persons.<br>-Active case searches were hardly being conducted by surveillance focal persons.<br>-Low documentation and incomplete case files of reported diseases<br>-Involvement of laboratories in surveillance activities at all levels was limited<br>-Not all outbreaks were properly investigated, lab confirmed and responded to appropriately.	III, A

							surveillance plans, and supervisory checklists e.t.c.)			
**23.**	Zambia	2012	Not specified	Haakonde et al.[23]	2018	To assess the factors affecting the implementation of the IDSR in public health care facilities in Rufunsa District, Zambia.	-A cross-sectional facility-based descriptive study<br>-Study conducted in all the nine Public Health Care Facilities located in Rufunsa District.<br>-Thirty-four participants were conveniently sampled from all the 9 health facilities<br>-Data collected using a pre-tested semi structured interviewer-administered questionnaires.	Core functions: Reporting, feedback<br><br>Support functions: Training, Supervision, Resources (logistical, financial, equipment)	-IDSR lacked local support in terms of periodical training and mentorship, regular and scheduled supervisory assistance and financial aid across surveillance levels.<br>-Lack of prompt feedback from higher levels.<br>-Lack of adequate coordination and communication across surveillance levels.	III, B
**24.**	Malawi	2014	Ebola, Tuberculosis, Malaria	Wu et al.[43]	2018	To describe the process of case identification and reporting in practice, and explore the differences between the IDSR guideline and actual implementation using timeliness and completeness as key	-The study adapted a mixed quantitative and qualitative approach<br>-Quantitative data was obtained from extracting IDSR monthly reporting rate summary data from national DHIS2<br>-Qualitative data was collected using key informant interviews and focus groups	Core functions: Case detection, Reporting<br><br>Support functions: Standard case definitions, Laboratory capacity, Training Supervision, Resources<br><br>Attributes: Reporting completeness and timeliness	-Differences between IDSR technical guideline and actual practice existed<br>-System shortcomings resulted from financial constraints and poor infrastructure	III, A

						indicators to evaluate IDSR performance in Malawi				
**25.**	Nigeria	2013	Cholera, Gastroenteritis, Measles, Typhoid fever, Schistosomiasis	Dairo et al.[19]	2018	To assess compliance with the surveillance and response guidelines for epidemic-prone diseases	-A cross sectional descriptive study<br>-Sample size: 198 disease surveillance officers<br>-Multi stage sampling was used to select the state, local government areas, health wards, health facilities and healthcare workers<br>-Data was collected using questionnaires, checklists, and records review of the monthly and weekly reporting surveillance data	Core functions: Case detection, Case confirmation, Case registration, Reporting, Feedback, Data analysis, Epidemic preparedness<br><br>Support functions: Standard case definitions, Supervision, Resources	-Inadequacy of laboratory support for surveillance activities at the local levels	III, B
**26.**	Madagascar	2013	Malaria, Diarrhoea, Acute Respiratory Infections, Measles, Acute Flaccid Paralysis, Chikungunya	Randriami-arana et al.[44]	2018	To evaluate performance of the reinforced integrated disease surveillance and response strategy using attributes and technological assessment	-Evaluation study design<br>-Health structures were stratified according to accessibility then stratified based on the type of basic healthcare centre followed by random sampling in each stratum<br>-Both face-to-face interviews and telephone interviews were used to collect data	Support functions: Standard and guidelines, resources (infrastructure)<br><br>Attributes: Simplicity, data quality, completeness and timeliness	-Short Message Service (SMS) improved IDSR data completeness but there is still a challenge with timeliness and data quality	III, A

**27.**	Uganda	2012	Cholera, Polio	Masiira et al.[45]	2019	To present findings from an assessment of IDSR core activities and support functions five years following implementation of the revitalized IDSR programme	-A cross sectional survey employing mixed quantitative and qualitative methods<br>-Quantitative data was collected using standardized questionnaires and checklists<br>-Focus group discussions and key informant interviews were used to gather qualitative data on different views related to IDSR implementation<br>-Two health districts were purposively sampled from 13 health regions<br>-Sample size: 202 health facilities and 606 health workers	Core functions: Case detection, Case registration, Case confirmation, Reporting, Feedback, Data analysis, Epidemic preparedness and response<br><br>Support functions: Standard case definitions, Laboratory capacity, Training, Resources,<br><br>Attributes: Reporting completeness and timeliness	-Inadequate number of trained frontline health workers<br>-Inadequate funding to support IDSR activities at district and health facility levels<br>-Poor perception of IDSR as a vertical programme by some health workers<br>-Irregular supervision<br>-High turnover of trained health workers	III, A

(a)**Evidence Levels:** Level I (Experimental studies, Randomized Controlled Trials); Level II (Quasi-experimental studies); Level III (Non-experimental studies)
(b)**Quality Grades:** A (High quality); B (Good Quality); C (Low Quality or major flaws)

Table 3. Summary of key study recommendations derived from the included studies

Surveillance functions	Authors	Publication year	Article title	Key study recommendations
**Recommendations on core functions**				
**Case confirmation**	Ngwa et al.[35]	2016	Cholera public health surveillance in the Republic of Cameroon-opportunities and challenges	Equipping health facilities and districts with surveillance personnel, computers and laboratories.<br>Equipping health facility laboratories to ensure early detection, confirmation and rapid response.
	Baghdadi[37]	2016	Assessment of core and support functions of case-based surveillance of meningitis in hospitals in Khartoum State in 2015	Formulating and distributing protocols on specimen (i.e. CSF) handling.
	Adokiya and Awoonor-Williams[38]	2016	Ebola virus disease surveillance and response preparedness in Northern Ghana	Improving laboratory capacity and prompt specimen taking.
	Dairo et al.[19]	2018	Compliance with epidemic-prone diseases surveillance and response guidelines among health officers at surveillance units in South-West Nigeria	Strengthening laboratory support for disease surveillance at the health facility level.
**Reporting**	Nnebue et al.[27]	2013	Effectiveness of data collection and information transmission process for disease notification in Anambra State, Nigeria	Provision of reporting forms and other logistics on regular basis.<br>Expanding sources of reporting.
	Lar et al.[24]	2015	Challenges of integrated disease surveillance response reporting among healthcare personnel in Mangu, Plateau State, Nigeria	Ensuring constant availability of IDSR reporting forms in the health facilities.
	Adokiya et al.(a)[21]	2015	Evaluation of the integrated disease surveillance and response system for infectious diseases control in northern Ghana	Addressing inconsistences between weekly and monthly surveillance data.

	Mwatondo et al.[36]	2016	Factors associated with adequate weekly reporting for disease surveillance data among health facilities in Nairobi County, Kenya, 2013	Providing urban settings with weekly reporting tools.
	Iwu et al.[25]	2016	Assessment of disease reporting among health care workers in a South Eastern State, Nigeria	Improved coordination, communication and support for disease reporting at local and state levels.
	Lakew et al.[42]	2017	Status of surveillance and routine immunization performances in Amhara Region, Ethiopia: findings from in-depth peer review	Improvement of surveillance documentation (i.e. copies of surveillance reports).
**Feedback**	Nnebue et al.[27]	2013	Effectiveness of data collection and information transmission process for disease notification in Anambra State, Nigeria	Ensuring adequate feedback of information.
	Abubakar et al.[29]	2013	Assessment of integrated disease surveillance and response strategy implementation in selected Local Government Areas of Kaduna State	Increased feedback from higher to lower levels.
	Nnebue et al.[26]	2014	Challenges of disease surveillance and notification in Anambra State, Nigeria	Promptly providing feedback within the disease surveillance and notification system.
	Begashaw and Tesfaye[28]	2016	Assessment of integrated disease surveillance and response implementation in special health facilities of Dawuro Zone	Improved feedback from higher to lower levels.
	Benson et al.[20]	2016	Survey of the perceptions of key stakeholders on the attributes of the South African Notifiable Diseases Surveillance System	Provision of feedback to all key stakeholders in the Notifiable Disease Surveillance System.
	Mboera et al.[30]	2017	Malaria surveillance and use of evidence in planning and decision making in Kilosa District, Tanzania	Providing feedback to motivate timely submission of reports.
**Data analysis**	Adokiya et al.[22]	2016	Evaluation of the reporting completeness and timeliness of the integrated disease surveillance and response system in northern Ghana	Initiation of plans to scale up data entry in district health information management systems at the periphery level to ensure data accuracy.

	Mboera et al.[30]	2017	Malaria surveillance and use of evidence in planning and decision making in Kilosa District, Tanzania	Capacity strengthening on data analysis and utilization at the facility and district levels.
	Lakew et al.[42]	2017	Status of surveillance and routine immunization performances in Amhara Region, Ethiopia: findings from in-depth peer review	Data analysis and surveillance performance indicators monitoring at the zonal and district (woreda) levels.
**Recommendations on support functions**				
**Training/Education**	Nnebue et al.[27]	2013	Effectiveness of data collection and information transmission process for disease notification in Anambra State, Nigeria	Periodic training and retraining of health personnel on disease surveillance and notification<br>Regular in-house training of health workers.
	Nnebue et al.[26]	2014	Challenges of disease surveillance and notification in Anambra State, Nigeria	Increased training for health workers on disease surveillance and notification.
	Issah et al.[34]	2015	Assessment of the usefulness of integrated disease surveillance and response on suspected Ebola cases in the Brong Ahafo Region, Ghana	Improved training activities for health personnel.
	Tsitsi et al.[33]	2015	Evaluation of the Notifiable Diseases Surveillance System in Beitbridge District, Zimbabwe 2015	Improve healthcare workers' knowledge on notifiable disease surveillance systems through training.
	Lar et al.[24]	2015	Challenges of integrated disease surveillance response reporting among healthcare personnel in Mangu, Plateau State, Nigeria	Train health personnel on correctly filling and compiling IDSR reports.
	Ngwa et al.[35]	2016	Cholera public health surveillance in the Republic of Cameroon-opportunities and challenges	Need for education and more supervision to ensure use of updated information and materials.<br>Trained surveillance personnel at the district level will be a great boost to the IDSR strategy.
	Adokiya et al.[22]	2016	Evaluation of the reporting completeness and timeliness of the integrated disease surveillance and response system in northern Ghana	Continued training of disease surveillance and health information officers to improve completeness, timeliness, data quality and accuracy of reporting.
	Benson et al.[20]	2016	Survey of the perceptions of key stakeholders on the attributes of the	Additional training to all key stakeholders in the Notifiable Disease Surveillance System.

			South African Notifiable Diseases Surveillance System	
	Mwatondo et al.[36]	2016	Factors associated with adequate weekly reporting for disease surveillance data among health facilities in Nairobi County, Kenya, 2013	Training on IDSR system.
	Baghdadi[37]	2016	Assessment of core and support functions of case-based surveillance of meningitis in hospitals in Khartoum State in 2015	Train laboratory and reporting unit health personnel.
	Mairosi et al.[39]	2016	Evaluation of notifiable disease surveillance system in Centenary District, Zimbabwe, 2016	Health workers to be trained on IDSR processes and the follow up actions.<br>Simplify training material to ease understanding and improve knowledge levels.
	Iwu et al.[25]	2016	Assessment of disease reporting among health care workers in a South Eastern State, Nigeria	Regular health staff training programs.
	Mandyata et al.[41]	2017	Challenges of implementing the integrated disease surveillance and response strategy in Zambia: a health worker perspective	Addressing the challenge of inadequately trained human resources.
	Haakonde et al.[23]	2018	Assessment of factors affecting the implementation of the integrated disease surveillance and response in public health care facilities: the case of Rufunsa District, Zambia	Ensure resources are secured and made available towards the provision of regular IDSR trainings targeting health care workers engaged in IDSR implementation.
	Randriamiarana et al.[44]	2018	Evaluation of the reinforced integrated disease surveillance and response strategy using short message service data transmission in two southern regions of Madagascar, 2014-15	Healthcare staff require training on IDSR guidelines and case definitions.
	Masiira et al.[45]	2019	Evaluation of integrated disease surveillance and response (IDSR) core and support functions after the revitalization of IDSR in Uganda from 2012 to 2016	Training of more health workers.<br>Regular IDSR training.<br>Incorporating IDSR training into the pre-service curriculum for health workers.<br>Training of community members in IDSR.
**Supervision**	Nnebue et al.[27]	2013	Effectiveness of data collection and information transmission process for	Improved supervision for surveillance data collection and transmission.

			disease notification in Anambra State, Nigeria	
	Nnebue et al.[26]	2014	Challenges of disease surveillance and notification in Anambra State, Nigeria	Ensuring adequate supervision.
	Tsitsi et al.[33]	2015	Evaluation of the Notifiable Diseases Surveillance System in Beitbridge District, Zimbabwe 2015	Support and supervision to ensure notifiable diseases are notified using the correct channels.
	Ngwa et al.[35]	2016	Cholera public health surveillance in the Republic of Cameroon- opportunities and challenges	Increased supervision to ensure use of updated information and materials.
	Lakew et al.[42]	2017	Status of surveillance and routine immunization performances in Amhara Region, Ethiopia: findings from in-depth peer review	Ensuring strict adherence to planned surveillance schedules (i.e. supervisory visits)
	Haakonde et al.[23]	2018	Assessment of factors affecting the implementation of the integrated disease surveillance and response in public health care facilities: the case of Rufunsa District, Zambia	Ensuring mentorship, regular and scheduled supervision is provided to strengthen IDSR implementation at the district level.
	Masiira et al.[45]	2019	Evaluation of integrated disease surveillance and response (IDSR) core and support functions after the revitalization of IDSR in Uganda from 2012 to 2016	Strengthening supervision to improve IDSR performance.
**Resources (human, financial, technical, logistical and infrastructure)**	Abubakar et al.[29]	2013	Assessment of integrated disease surveillance and response strategy implementation in selected Local Government Areas of Kaduna State	Provision of sufficient logistical resources and data management tools.
	Nnebue et al.[27]	2013	Effectiveness of data collection and information transmission process for disease notification in Anambra State, Nigeria	Improved funding for disease surveillance activities.
	Nnebue et al.[26]	2014	Challenges of disease surveillance and notification in Anambra State, Nigeria	Provision of transportation, adequate supply of reporting forms and other logistics.
	Adokiya et al.(a)[21]	2015	Evaluation of the integrated disease surveillance and response system for infectious diseases control in northern Ghana	Ensuring adequate support for and communication within the IDSR system.

Tsitsi et al.[33]	2014	Evaluation of the Notifiable Diseases Surveillance System in Beitbridge District, Zimbabwe 2015	Distribution of notification forms to all health facilities. Engaging telecommunication service providers to put up network boosters.
Ngwa et al.[35]	2016	Cholera public health surveillance in the Republic of Cameroon- opportunities and challenges	Equipping health facilities and districts with surveillance personnel, computers and laboratories. Computers and trained surveillance personnel at the district level will boost the IDSR strategy.
Mwatondo et al.[36]	2016	Factors associated with adequate weekly reporting for disease surveillance data among health facilities in Nairobi County, Kenya, 2013	Designation of a dedicated surveillance focal person. Availing posters and guidelines on IDSR functions to improve weekly reporting.
Iwu et al.[25]	2016	Assessment of disease reporting among health care workers in a South Eastern State, Nigeria	Adequate and equitable funding for the disease reporting process including regular staff remuneration.
Begashaw and Tesfaye[28]	2016	Assessment of integrated disease surveillance and response implementation in special health facilities of Dawuro Zone	Ensuring sufficient surveillance resources are provided in health facilities.
Mboera et al.[30]	2017	Malaria surveillance and use of evidence in planning and decision making in Kilosa District, Tanzania	Strengthening the technical capacity of health facility, district and national levels on all aspects of health information systems.
Mandyata et al.[41]	2017	Challenges of implementing the integrated disease surveillance and response strategy in Zambia: a health worker perspective	Addressing the challenge of poor infrastructure, coordination, lack of provision of optimal technical support to DHMTs and health facilities.
Dairo et al.[19]	2018	Compliance with epidemic-prone diseases surveillance and response guidelines among health officers at surveillance units in South-West Nigeria	Ensuring funds provision and other resources to surveillance workers to achieve effective disease control.
Haakonde et al.[23]	2018	Assessment of factors affecting the implementation of the integrated disease surveillance and response in public health care facilities: the case of Rufunsa District, Zambia	Allocating funds to support IDSR activities in the health sector budget.
Masiira et al.[45]	2019	Evaluation of integrated disease surveillance and response (IDSR) core and support functions after the revitalization of IDSR in Uganda from 2012 to 2016	Increasing IDSR funding at district and health facility levels.

**Recommendations on surveillance attributes**				
**Timeliness and completeness**	Nnebue et al.[27]	2013	Effectiveness of data collection and information transmission process for disease notification in Anambra State, Nigeria	Increased awareness on importance of effective reporting.
	Maponga et al.[31]	2014	Evaluation of the notifiable diseases surveillance system in Sanyati district, Zimbabwe, 2010-2011	Availing information on when diseases are being notified.
	Issah et al.[34]	2015	Assessment of the usefulness of integrated disease surveillance and response on suspected Ebola cases in the Brong Ahafo Region, Ghana	Improved timely reporting of notifiable conditions.
	Adokiya et al.[22]	2016	Evaluation of the reporting completeness and timeliness of the integrated disease surveillance and response system in northern Ghana	Consistency during weekly and monthly reporting. Conducting further investigations to address reporting completeness.
	Mairosi et al.[39]	2016	Evaluation of notifiable disease surveillance system in Centenary District, Zimbabwe, 2016	Provision of time information on disease notification.
	Benson et al.[20]	2016	Survey of the perceptions of key stakeholders on the attributes of the South African Notifiable Diseases Surveillance System	Health reforms to address surveillance system reporting timeliness.
	Ngwa et al.[35]	2016	Cholera public health surveillance in the Republic of Cameroon-opportunities and challenges	Enhancing human resource capacity.
	Mboera et al.[30]	2017	Malaria surveillance and use of evidence in planning and decision making in Kilosa District, Tanzania	Awareness on specific reporting dates.
	Randriamiarana et al.[44]	2018	Evaluation of the reinforced integrated disease surveillance and response strategy using short message service data transmission in two southern regions of Madagascar, 2014-15	Reducing workload, increasing training and improving mobile network infrastructure.
	Masiira et al.[45]	2019	Evaluation of integrated disease surveillance and response (IDSR) core and support functions after the	Enhanced IDSR training and adopting mobile-based reporting.

			revitalization of IDSR in Uganda from 2012 to 2016	
**Data Quality/Accuracy**	Maponga et al.[31]	2014	Evaluation of the notifiable diseases surveillance system in Sanyati district, Zimbabwe, 2010-2011	Ensuring complete and precise reporting.
	Mairosi et al.[39]	2016	Evaluation of notifiable disease surveillance system in Centenary District, Zimbabwe, 2016	Reducing missed data occurrences.
	Adokiya et al.[22]	2016	Evaluation of the reporting completeness and timeliness of the integrated disease surveillance and response system in northern Ghana	Adopting the DHIS2 reporting system starting from the peripheral level.
	Randriamiarana et al.[44]	2018	Evaluation of the reinforced integrated disease surveillance and response strategy using short message service data transmission in two southern regions of Madagascar, 2014-15	Providing quality control mechanisms to avoid transmission of erroneous data.
**Usefulness**	Benson et al.[20]	2016	Survey of the perceptions of key stakeholders on the attributes of the South African Notifiable Diseases Surveillance System	Health reforms to encourage use of surveillance data.
	Mairosi et al.[39]	2016	Evaluation of notifiable disease surveillance system in Centenary District, Zimbabwe, 2016	Documenting public health action reports based on surveillance data.
**Acceptability**	Maponga et al.[31]	2014	Evaluation of the notifiable diseases surveillance system in Sanyati district, Zimbabwe, 2010-2011	Provision of clear job descriptions.
	Tsitsi et al.[33]	2015	Evaluation of the Notifiable Diseases Surveillance System in Beitbridge District, Zimbabwe 2015	Aligning surveillance activities with day-to-day duties.
	Mairosi et al.[39]	2016	Evaluation of notifiable disease surveillance system in Centenary District, Zimbabwe, 2016	Designation of surveillance focal persons.
**Stability**	Maponga et al.[31]	2014	Evaluation of the notifiable diseases surveillance system in Sanyati district, Zimbabwe, 2010-2011	Availability of human resources.
	Tsitsi et al.[33]	2015	Evaluation of the Notifiable Diseases Surveillance System in Beitbridge District, Zimbabwe 2015	Availing material resources.

	Mairosi et al.[39]	2016	Evaluation of notifiable disease surveillance system in Centenary District, Zimbabwe, 2016	Enhanced training and provision of communication and logistical facilities.
	Mandyata et al.[41]	2017	Challenges of implementing the integrated disease surveillance and response strategy in Zambia: a health worker perspective	Improved internet connectivity and infrastructure.
	Benson et al.[40]	2017	Comparing laboratory surveillance with the notifiable diseases surveillance system in South Africa	Provision of reliable diagnostic equipment.
**Simplicity**	Maponga et al.[31]	2014	Evaluation of the notifiable diseases surveillance system in Sanyati district, Zimbabwe, 2010-2011	Ease of completing notification forms.
	Tsitsi et al.[33]	2015	Evaluation of the Notifiable Diseases Surveillance System in Beitbridge District, Zimbabwe 2015	Reduced length of time required to complete notification forms.
	Mairosi et al.[39]	2016	Evaluation of notifiable disease surveillance system in Centenary District, Zimbabwe, 2016	Ease of understanding surveillance system functionalities.
	Benson et al.[20]	2016	Survey of the perceptions of key stakeholders on the attributes of the South African Notifiable Diseases Surveillance System	Simplification of surveillance system at operational level.
	Randriamiarana et al.[44]	2018	Evaluation of the reinforced integrated disease surveillance and response strategy using short message service data transmission in two southern regions of Madagascar, 2014-15	Distribution and display of simplified and understandable terms of reference and case definition guidelines.
**Further recommendations**				
**Alternative surveillance strategies**	Maponga et al.[31]	2014	Evaluation of the notifiable diseases surveillance system in Sanyati district, Zimbabwe, 2010-2011	Need for the Ministry of Health to develop an electronic based system for surveillance data reporting riding on the availability of mobile phone use.
	Tsitsi et al.[33]	2015	Evaluation of the Notifiable Diseases Surveillance System in Beitbridge District, Zimbabwe 2015	Adoption of an electronic/mobile channel in notifying diseases to cut down on costs of the paper-based system.
	Issah et al.[34]	2015	Assessment of the usefulness of integrated disease surveillance and response on suspected Ebola cases in the Brong Ahafo Region, Ghana	Improving and focusing on community based surveillance system by bringing it into the mainstream surveillance for Ebola Viral Disease.

	Ngwa et al.[35]	2016	Cholera public health surveillance in the Republic of Cameroon-opportunities and challenges	Equipping all health facilities with the ‘green line’ mobile surveillance approach.
	Benson et al.[20]	2016	Survey of the perceptions of key stakeholders on the attributes of the South African Notifiable Diseases Surveillance System	Introducing an electronic system including use of mobile telephone technology to address perceived weaknesses of the NDSS.
	Lakew et al.[42]	2017	Status of surveillance and routine immunization performances in Amhara Region, Ethiopia: findings from in-depth peer review	Properly formulating operational plans to improve active case search with realistic prioritization of visiting reporting sites.
	Wu et al.[43]	2018	Integrated Disease Surveillance and Response (IDSR) in Malawi: Implementation gaps and challenges for timely alert	Improved technology infrastructure and adapting mobile technologies.<br>Utilization of syndromic surveillance approach combined with systematic virological testing.
	Randriamiarana et al.[44]	2018	Evaluation of the reinforced integrated disease surveillance and response strategy using short message service data transmission in two southern regions of Madagascar, 2014-15	Improved data collection, compilation and transfer through an electronic system.<br>Increasing mobile network coverage.
**Further research**	Maponga et al.[31]	2014	Evaluation of the notifiable diseases surveillance system in Sanyati district, Zimbabwe, 2010-2011	Need for further research on the effect of training health-care workers on the surveillance system.
	Adokiya et al.[22]	2016	Evaluation of the reporting completeness and timeliness of the integrated disease surveillance and response system in northern Ghana	Further research to improve reporting completeness and timeliness of surveillance data.
	Mwatondo et al.[36]	2016	Factors associated with adequate weekly reporting for disease surveillance data among health facilities in Nairobi County, Kenya, 2013	Conducting further studies in rural or mixed settings in different Kenyan counties in order to gather information on the challenges of reporting in health facilities.
**Other recommendations**	Adokiya et al. 2015(b)[32]	2015	The integrated disease surveillance and response system in Northern Ghana: challenges to the core and support functions	Need to recognize disease surveillance activities as essential for the overall functioning of the health system.

Core functions

Case Confirmation

Case confirmation efforts within surveillance systems to inform prompt public health action requires enhanced laboratory capacity [19]. Laboratory services absence in health facilities (HFs) to confirm cholera cases and outbreaks was reported in Cameroon [35]. Therefore, this required laboratory facilities and equipment be provided to ensure prompt detection, confirmation and response to cholera cases [35]. Similarly, laboratory capacity strengthening and prompt specimen collection was recommended in Ghana to ensure adequate surveillance and response preparedness to Ebola [38]. Functions relating to case confirmation were absent in HF[illegible] in Khartoum State, hence necessitating need to formulate and distribute protocols for specimen handling specific to meningitis [37].

Reporting

Studies have reported lack of specific forms for reporting notifiable diseases [25-27, 31, 33]. Poor reporting performance in Zimbabwe was due to absence of disease notification forms and reporting guidelines [31]. Furthermore, a study in Kenya reported having weekly reporting forms present in HFs significantly increased disease surveillance reporting odds [36]. Therefore, availing IDSR reporting tools would ensure continuity and consistency in reporting surveillance data [24, 36]. In Ghana, inaccuracies and missing data in surveillance reports submitted from peripheral to regional level resulted from uncertainties on the most appropriate reporting channel [21]. This required addressing inconsistencies of weekly and monthly reports submitted through the various channels [21]. Advocating for improvements and clarity on the proper reporting channels would avoid frequent communication breakdowns and missing data in surveillance reports [21]. Improved

surveillance documentation was recommended since most regional surveillance offices lacked active case searches written reports from reporting sites in Ethiopia [42].

Feedback

Feedback to HFs from regional level was mainly through supervisory visits and reported cases investigations as opposed to written feedback [31, 33]. Low feedback was reported from higher levels to both public and private HFs [24, 29, 31]. Inadequate feedback to HFs may demotivate health workers (HWs), limiting their efforts towards efficient and timely reporting [24, 30]. Similarly, prompt feedback on disease reports sent to higher levels was lacking in Zambia, hence HWs felt this compromised efforts to limit disease transmission [23]. HWs low perceptions on the disease surveillance system's acceptability, flexibility and usefulness would be resolved through sufficient feedback [20]. Adequate feedback provision to motivate HWs to submit timely reports for malaria cases would address inefficient disease surveillance reporting in Tanzania [30]. Similarly, improved feedback from higher to lower levels would motivate health staff to report efficiently and influence their performance in surveillance activities as reported in Nigeria and Ethiopia [28, 29]. Furthermore, ensuring adequate and prompt feedback within disease surveillance and notification system would alleviate major challenges faced within the system [26, 27].

Data Analysis

Data transmission challenges using paper-based reporting from periphery to district level increased error introduction likelihood in the reported data [22]. Hence, necessitating plans initiation for scaling up data entry in DHIMS2 at the periphery level to improve data accuracy in Ghana [22]. Similarly, challenges involving limited capacity and low evidence of proper data analysis at the hospital and district levels in Tanzania were to be mitigated by strengthening capacity for data

analysis and availing tallying sheets, register books and reporting forms [30]. Limited use of outcomes from surveillance performance analysis in Ethiopia required an undertaking to analyse surveillance data and closely monitor surveillance performance indicators at regional levels [42].

Support functions

Training

Low knowledge on correct forms for reporting notifiable diseases negatively affected timely disease reporting in Zimbabwe [33]. Therefore, HWs required training to improve their knowledge on notifiable disease surveillance systems through induction and on job training [33]. Improved reporting practices as a result of forms availability and recognising HWs' reporting efforts was significantly associated with a post-training intervention in Nigeria [24]. Consequently, continued health worker training on correct form filling and reports compilation was recommended [24]. In Ghana, formal IDSR training with a focus on detection and reporting of Ebola Viral Disease (EVD) suspected cases was lacking at HF and community levels [34]. Hence, an integrated and sustained funding support towards HP training at facility and community levels would ensure effective EVD suspected cases contact tracing and halt disease transmission [34]. Disease surveillance training especially at community, HF and district levels was limited in comparison to training undertaken at the regional and national levels in Cameroon [35]. The mitigation measure recommended was to increase HP training at district level to enhance IDSR strategy implementation [35]. Moreover, to mitigate insufficient reporting completeness and timeliness coupled with poor quality surveillance data in Ghana, it required continued training of both disease surveillance and health information officers [22]. Similarly, inadequate training was perceived to limit disease reporting, hence requiring regular health staff training [25]. Additional training for notifiable disease surveillance stakeholders would influence their willingness to participate across all system levels

[20]. This would further influence system acceptability and the value stakeholders attach to the system [20].

IDSR training was inadequate in Zambia resulting in HWs dependence on prior knowledge while executing their duties [41]. Therefore, they required adequate training to improve the quality and quantity of surveillance data being generated and utilised for decision-making [41]. HWs training needs on IDSR system aspects needed addressing to enable proper identification of designated focal surveillance persons in Kenya [36]. In South Sudan, increased HP training in hospital reporting units and laboratories would improve meningitis case-based reporting within the surveillance system [37]. Although HWs in Zimbabwe perceived notifiable disease surveillance system to be simple and easy to use, training was necessary to increase their understanding of IDSR processes and follow up actions. This would be achieved through simplifying training materials to ease understanding of the system [39]. In Zambia, most respondents felt securing adequate funds to conduct periodical training and re-training could strengthen all IDSR system implementation aspects [23]. Similarly, challenges associated with inadequate training in Nigeria was to be mitigated through regular in-house training and re-training of HP on disease surveillance and notification [26, 27]. In Uganda, having an inadequate number of frontline HP trained on IDSR system was to be resolved through IDSR training incorporation in health worker’s initial pre-service curriculum and community involvement in training [45].

Supervision

Enhanced surveillance supervisory efforts at HF level would ensure notifiable diseases are notified through correct channels [33]. However, most supervisory reviews only focused on surveillance activities involving immunisable diseases, tuberculosis and HIV/AIDs [31, 33]. Similarly, partial supervision was undertaken in Cameroon at regional and district levels, while at community and

HF levels there was complete absence of supervisory activities [35]. Increased awareness on supervision benefits and efforts to enhance supervision would ensure utilisation of up-to-date surveillance information and materials amongst HCWs [35]. Surveillance focal persons irregularly provided supportive supervision for active case searches in Ethiopia, hence requiring strict adherence to planned surveillance schedules for conducting supervisory visits [42]. Furthermore, HWs at the district level in Zambia felt regular and scheduled supervisory assistance from higher levels would strengthen the IDSR system [23]. In addition, increased supervision was required to ensure disease notification system were effective in data collection and information transmission in Nigeria [27]. Irregular supervision was still an existing challenge in the revitalised IDSR programme that required addressing to improve IDSR performance in Uganda [45].

Resources

Surveillance data analysis and management tools unavailability at HF and district levels was reported in Cameroon [35]. Hence, requiring HFs and district levels to be equipped with computers [35]. Similarly, data management tools availability was to be complemented by their functionality to ease surveillance data entry and analysis [28, 29]. On the other hand, the main challenge facing cellphone communication reporting channels was poor network infrastructure in Zimbabwe [33]. Hence, requiring telecommunication service providers engagement to set up network boosters to improve communication and timely reporting [33]. Limited utilisation of routine health information for performance monitoring was to be mitigated by ensuring health information systems strengthening at all surveillance levels in Tanzania [30]. Notification forms unavailability in Zimbabwe hindered HCWs efforts for disease notification and delayed epidemic investigations [33]. Therefore, it required notification forms are distributed to all HFs [33]. In Kenya, HFs displaying visual aids for IDSR functions were more likely to report surveillance data [36]. Hence,

to strengthen these efforts it was recommended that posters and guidelines on IDSR functions are provided to improve reporting [36]. In Zambia, IDSR technical guidelines were unavailable in HFs, hence they lacked the appropriate procedures for handling suspected cases of notifiable diseases [41]. This identified the need for technical support especially at HF levels to promote and improve early disease detection [41].

Health facilities lacking HWs designated to manage disease surveillance data had decreased odds of adequate reporting [36]. Hence, designating a surveillance focal person in HFs would improve surveillance reporting [36]. Likewise, there was need to equip the district and HF levels with trained surveillance personnel in Cameroon [35]. Healthcare personnel identified lack of financial aid as a hindrance to IDSR implementation in Zambia. Hence, necessitating funds allocation in the health sector budget to support IDSR activities [23]. In Nigeria, improved funding would ensure effective surveillance data collection and transmission process [27]. Furthermore, in the South Eastern State of Nigeria, adequate and equitable funding was required to facilitate the disease reporting process [25]. Similarly, increased IDSR funding was recommended to support surveillance activities at the district and HF levels in Uganda [45].

Surveillance attributes

Key recommendations on improving the surveillance attributes were specific to reporting timeliness and completeness, data quality and accuracy, usefulness, acceptability, simplicity and stability of the surveillance system [20, 22, 30, 31, 33, 35, 39-41, 43-45].

Timeliness and completeness

An under-performing surveillance quality function requiring improvement was lack of timely reporting within the 24 hour window period for Ebola suspected cases at the regional surveillance

unit in Ghana [34]. Similarly, inconsistencies in weekly and monthly reporting timeliness were observed in northern Ghana, hence requiring an urgent need to strengthen the disease surveillance system to enable rapid response to infectious disease outbreaks [22]. Information unavailability on disease notification from HFs to district level, negated efforts to ascertain surveillance data timeliness in Zimbabwe [31, 39]. Similarly, HCWs in Tanzania responsible for malaria surveillance data reporting were unaware of specific dates when reports were submitted from HFs to the next reporting level [30]. Therefore, information provision bearing specific reporting dates would be critical to determining surveillance system reporting timeliness. Improved reporting timeliness and completeness in Uganda resulted from enhanced IDSR training, which created increased disease surveillance awareness amongst health providers coupled with mobile-SMS based reporting [45]. Similarly in Malawi, adapting an electronic reporting system and mobile technologies would mitigate disease notification challenges from HFs to the next level [43]. Furthermore, increased awareness on effective reporting would resolve reporting reluctance amongst HWs and improve reporting timeliness in Nigeria [27]. Reduced workload, enhanced training and improved mobile infrastructure would improve reporting completeness and timeliness according to medical and paramedical agents in Madagascar [44]. Moreover, few healthcare providers in South Africa agreed the existing notifiable disease surveillance system (NDSS) provided timely information to prompt action. Hence, there were calls for future reforms to give priority to 'timeliness' attribute of South African NDSS to ensure effective disease outbreak containment [20]. Variations in reporting completeness across health system levels in northern Ghana and Cameroon were to be mitigated through undertaking further research investigations to address the root causes and enhancing human resource capacity respectively [22, 35].

Data quality and accuracy

Scaling-up data entry into the District Health Information Management System (DHIMS2) starting from the health system periphery level would address data quality and accuracy concerns [22]. Erroneous data transmission across surveillance system undermined data quality and surveillance data reliability [44]. Likewise, ensuring missing data in disease notification forms occurred less frequently would improve reported data quality [31, 39]. Moreover, data entries completeness and correctness in notification forms was a data quality measure in Zimbabwe [39].

Simplicity

Availing easily understandable and simplified terms of reference and case definitions would ease surveillance activities in Madagascar [44]. Notifiable disease surveillance system evaluation in Zimbabwe identified ease and duration of completing disease notification forms as a determinant of system's simplicity [31, 33, 39]. HWs' positive perceptions on simplicity of the system motivates their involvement in notifying diseases [39]. In contrast, healthcare stakeholders involved at operational level of the surveillance system in South Africa perceived the system to be complex compared to their counterparts at health management level. Hence, identifying need for simplifying the system at disease detection and response level [20].

Usefulness

There were calls for reforms of South African NDSS to encourage surveillance data use by healthcare providers for outbreak response and communicable diseases control [20]. Besides, effective reports documentation on public health actions or decisions following data collected through disease surveillance systems would be vital in assessing system's usefulness [39].

Acceptability

To resolve HWs' lack of willingness to notify diseases in Zimbabwe, there was need for clear designation of surveillance focal persons within HFs [39]. Similarly, HWs willingness to

participate in surveillance activities was influenced by disease surveillance being in line with their job description [31, 33]. Calls for reforms of South African NDSS aimed to improve system's acceptability amongst healthcare personnel [20].

Stability

Enhanced stability of the existing surveillance system in Zimbabwe required an increased number of staff are trained on disease surveillance and provision of functional communication equipment and transport facilities [39]. Similarly, reports on surveillance systems evaluation in Zimbabwe assessed system's stability based on human and material resource availability [31, 33]. Improved stability of NDSS in South Africa implied the system should be able to provide reliable diagnostic results on notifiable diseases [40]. Furthermore, surveillance system stability in Zambia was dependent on internet connectivity consistency or internet outages frequency for a specific time during reporting [41].

Alternative surveillance strategies

In studies conducted in Zimbabwe, the reporting process was cost intensive due to transport expenses incurred while submitting paper-based disease notification forms. Therefore, electronic-based system adoption for reporting within HFs would minimise these costs [33, 39]. Disease notifications using the paper-based system for sending notification forms was cost intensive. Hence, requiring the establishment of an electronic-based system for surveillance data reporting and mobile phone technology utilisation [31]. Similarly, there was need to equip HFs with mobile phone surveillance to effectively capture cholera cases in Cameroon [35]. A suspected Ebola outbreak in Ghana necessitated community-based disease surveillance revival as an active mainstream surveillance system to effectively detect and monitor suspected diseases [34]. In

Ethiopia, operational plans formulation for conducting prioritised surveillance visits to specific reporting sites would improve active case searches [42]. To resolve discerned weaknesses in attributes of NDSS in South Africa, it was important to establish an electronic surveillance system utilising mobile phone technology [20]. Similarly, adopting mobile technologies in addition to utilising syndromic surveillance approaches were recommended to strengthen IDSR system in Malawi [43].

Further research on surveillance

Inconsistencies in surveillance data completeness and timeliness in northern Ghana necessitated further research to mitigate this shortcoming [22]. Limited knowledge amongst HWs on the NDSS coupled with its sub-optimal performance was reported in Zimbabwe [31]. The resolution reached was to initiate further research efforts to assess the effect of health worker training on surveillance system performance [31]. In Kenya, further studies to establish reporting challenges facing HFs in remote settings were recommended [36]. Similarly, there was need to undertake further research in Zimbabwe to ascertain the effect of training HWs on surveillance system aspects [31].

Noteworthy, sub-themes frequency effect size summary based on how often a particular sub-theme appeared in the body of literature reviewed indicated sub-themes relating to timeliness and completeness; knowledge on surveillance system; technical guidelines, materials and logistics; and electronic-based surveillance as the predominant emerging sub-themes with frequency effect sizes of 37%, 29.6%, 22.2% and 22.2% respectively (Table 3). On the other hand, intensity effect size of studies based on how much each study contributes, in terms of the number of sub-themes it includes to the overall body of literature reviewed indicated articles authored by Maponga et al.

2016, Tsitsi et al. 2015, Ngwa et al. 2016, and Mairosi et al. 2016 contributed significantly to the reviewed literature [31, 33, 35, 39].

Discussion

The reviewed assessments studies clearly indicate milestones achieved since adopting the revised IDSR guidelines in Africa, in comparison to findings from a previous review [9]. However, the current review still identifies persistent challenges in IDSR system implementation. The review identified key recommendations to alleviate existing IDSR system challenges based on surveillance functions and these were categorised into specific sub-themes (Table 4). The recommendations aimed to influence existing health policies by strengthening IDSR system critical functions parallel to reinforcing core surveillance capacities laid out in the IHR [3].

Table 4. Summary of sub-themes with frequency and intensity effect sizes

Main Themes	Sub-themes	FES (%) ↓	Nnebue et al . 2013[27]	Abubakar et al. 2013[29]	Maponga et al. 2014[31]	Nnebue et al. 2014[26]	Adokiya et al. 2015 (a)[21]	Adokiya et al. 2015 (b)[32]	Tsitsi et al. 2015[33]	Issah et al. 2015[34]	Lar et al. 2015[24]	Ngwa et al. 2016[35]	Benson et al. 2016[20]	Mwatondo et al. 2016[36]	Adokiya et al. 2016[22]	Baghdadi , 2016[37]	Adokiya and Awoonor-Wiliams, 2016[38]	Mairosi et al. 2016[39]	Iwu et al. 2016[25]	Begashaw and Tesfaye, 2016[28]	Benson et al. 2017[40]	Mandyata et al. 2017[41]	Mboera et al. 2017[30]	Lakew et al. 2017[42]	Haakonde et al. 2018[23]	Wu et al. 2018[43]	Dairo et al. 2018[19]	Randriamiarana et al. 2018[44]	Masiira et al. 2019[45]
**IES (%)**	→		**13.8**	**3.4**	**34.5**	**6.9**	**6.9**	**6.9**	**31.0**	**10.3**	**6.9**	**24.1**	**17.2**	**17.2**	**13.8**	**10.3**	**6.9**	**24.1**	**6.9**	**6.9**	**3.4**	**10.3**	**13.8**	**10.3**	**10.3**	**6.9**	**3.4**	**17.2**	**13.8**
**Surveillance Core Functions**	***Case Confirmation***																												
	Provision of laboratory facilities	**7.4**										X					X												
	Improved specimen handling	**3.7**														X													
	Reporting																												
	Provision of reporting forms	**14.8**			X				X		X			X															
	Reporting quality	**7.4**						X								X													
	Feedback																												

	Improved feedback from higher to lower levels	**11.1**		X															X			X							
	Improved health workers' attitudes	**3.7**											X																
	Data Analysis																												
	Improved data accuracy and quality	**7.4**													X								X						
	Surveillance performance monitoring	**3.7**																						X					
**Surveillance Support Functions**	***Training***																												
	Knowledge on surveillance systems	**29.6**	X			X			X	X				X				X										X	X
	Improved surveillance data quality	**18.5**					X				X					X			X			X							
	Improved performance of the surveillance system	**11.1**			X							X													X				
	Supervision																												
	Identification of correct reporting channels	**7.4**			X				X																				
	Utilisation of up-to-date information	**3.7**										X																	

	Strengthening the surveillance system	**14.8**	X																					X	X				X
	Resources																												
	Equipment and Infrastructure	**14.8**					X		X			X										X							
	Technical guidelines, materials and logistics	**22.2**				X			X					X						X			X				X		
	Human resources	**7.4**										X		X															
	Financial resources	**18.5**	X																X					X	X				X
**Surveillance Attribute Functions**	Timeliness and completeness	**37.0**	X		X					X		X	X		X			X					X					X	X
	Data Quality/Accuracy	**14.8**			X										X			X										X	
	Usefulness	**7.4**											X					X											
	Acceptability	**11.1**			X				X									X											
	Stability	**18.5**			X				X									X			X	X							
	Simplicity	**18.5**			X				X				X					X										X	

**Further recommendations**	***Alternative surveillance strategies***																												
	Electronic-based surveillance	**22.2**			X				X			X	X													X		X	
	Community-based surveillance	**3.7**								X																			
	Syndromic surveillance	**3.7**																								X			
	Further research	**11.1**			X									X	X														
	Other recommendations	**7.4**						X									X												

X - denotes presence of a given finding; **FES (Frequent Effect Size)** – how often a particular sub-theme appeared in the reviewed literature; **IES (Intensity Effect Size) -** how much each included study contributes to the reviewed literature based on number of the emerging sub-themes.

A review of recommendations ensuing from previous studies focused on assessing IDSR system functions aided in generating a matrix of major themes derived from study recommendations. These recommendations were inclined to specific surveillance core, support and attribute functions. Core functions included; case confirmation, reporting, data analysis and feedback. Surveillance support functions included; training, supervision and resources. Surveillance attributes included; reporting timeliness and completeness, data quality and accuracy, usefulness, acceptability, simplicity and stability. Other key recommendations alluded to alternative surveillance strategies and calls for further research. Main themes were based on surveillance functions and sub-themes derived from emerging recommendations. Surveillance core functions themes and sub themes provided in parentheses were; case confirmation (laboratory facilities provision and improved specimen handling); reporting (reporting forms provision and reporting quality); feedback (improved feedback from higher to lower levels and improved HWs' attitudes); data analysis (data accuracy, data quality and surveillance performance monitoring). On the other hand, key recommendations regarding surveillance support functions were; training (surveillance systems knowledge, improved surveillance data quality and system performance); supervision (correct reporting channels identification, up-to-date information utilisation and surveillance system strengthening); resources (equipment, infrastructure, technical guidelines and materials, human resources, financial resources). Recommendations regarding surveillance attributes were specific to reporting timeliness and completeness, data quality and accuracy, usefulness, acceptability, simplicity and stability. Other recommendations included; alternative surveillance strategies (electronic based surveillance, community based surveillance and syndromic surveillance) and further research on surveillance.

Identifying need to confirm cases is paramount to informing effective and prompt action to avoid disease outbreaks. Therefore, strengthening case confirmation efforts through providing fully functional and adequately equipped laboratory facilities right from the peripheral level is critical for surveillance system effectiveness. IDSR implementation consolidates surveillance efforts with laboratory support to achieve effective public health action and response [34]. Improving IDSR strategy performance requires laboratory capacity strengthening to promptly confirm epidemic-prone diseases [34]. Limited protocols provision to guide specimen collection and handling was evidenced in HFs in South Sudan. Similarly, lack of standard operating procedures for specimen collection, packaging and transportation in hospitals in Ghana limited case confirmatory efforts [34]. Laboratory capacity challenges still exist in Africa despite the progress made in complying with IDSR and IHR requirements [46]. In South Africa, the need for a legislative requirement to oblige all laboratories to notify all conditions would resolve challenges within the NDSS [40]. Therefore, enhanced laboratory capacity in providing timely collection, transportation and prompt discharge of results is vital to IDSR system functioning in Africa [34]. A key policy challenge relating to laboratory capacity among African countries is lack of ownership and consideration of laboratory undertakings and budgets in national health plans [46]. Hence, limiting resource mobilisation and sustainability of laboratory capacity.

Effective surveillance data reporting through correct channels, using appropriate methods and in a timely manner is vital in any given disease surveillance system. IDSR implementation in Africa is still being confronted with reporting challenges especially at HF level, which is characterised by limited generation of reliable health information [25]. Reporting forms and guidelines unavailability has an impact on HWs reporting performance and impedes their ability to conduct outbreak investigations [31]. In addition, HWs' awareness on the link between their day-to-day

activities and disease reporting will improve their willingness to adhere to reporting guidelines [25, 33]. Similarly, reporting forms and guidelines availability in HFs increases disease reporting likelihood [36]. Simplified reporting systems and procedures motivates HWs to report surveillance data effectively as opposed to complex systems [31]. Surveillance data under reporting is associated with severe consequences as result of increased disease burden [47]. Frequent data quality checks through constant supervision would ensure quality surveillance data reporting to the next levels to inform appropriate public health action [31]. Furthermore, reporting forms missing critical information might upset data analysis efforts and further investigations [31]. Hence, the overall surveillance data quality reported dictates public health response quality.

Feedback is an essential surveillance function and a core IDSR indicator in measuring system's performance [48]. Feedback within a surveillance system involves verbal or written communication from a higher to a lower level. Verbal feedback to HFs is a common practice by HP in Africa usually through half year or quarterly meetings [21, 31, 35]. However, rarely are written feedback bulletins issued at HF levels. This identifies a major challenge in feedback mechanisms of existing surveillance systems in Africa, which neglect peripheral levels. Feedback from higher to lower surveillance levels is generally low according to HWs in both public and private facilities [31]. Lower surveillance levels are commonly discriminated in receiving epidemiological bulletins from the national levels in comparison to regional surveillance levels [35]. Feedback on reported data influences HWs attitudes and willingness to participate in surveillance activities [20, 30, 31]. HWs are motivated to engage in surveillance undertakings when they receive feedback correcting their mistakes and improving their knowledge on the surveillance system [31]. Notably, surveillance data forwarding to higher levels with hardly any

feedback received at lower levels was commonly practiced prior to revised IDSR guidelines adoption with no clear feedback mechanisms [49, 50].

Surveillance data collection, analysis, interpretation and utilisation are intended to inform public health action [48]. Surveillance data analysis involves morbidity and mortality data aggregation to quantify disease burden at a particular time in a given region. Consequently, this analysis is utilised to monitor disease trends to inform case-based investigations and response [35]. Minimal and basic data analysis is a common practice in HFs with little or no documented evidence of analysed data [30]. This result from misguided perceptions amongst HCWs on surveillance data generation purposes only for onward reporting rather than utilisation at HF level [19]. Few HP initiated data analysis efforts at HF level in the African region, with less than fifty percent of surveillance personnel involved in data analysis [19, 29, 48, 51]. Moreover, low knowledge amongst HWs on analysis guidelines provided in IDSR manuals contributes to insufficient data analysis [30]. Lack of structured data analysis frameworks at various surveillance levels pose a challenge to accomplishing IDSR goals [35]. However, district health management information systems (DHIMS2) adoption by African countries has the potential to enhance surveillance data analysis [21]. Even though the DHIMS2 still faces data accuracy challenges resulting from difficulties of validating information reported to the system through various channels [21]. Limited data analysis capacity and quality in Africa can be attributed to insufficient provision of data management tools including computers, reporting forms, registers and tally sheets [30].

Low knowledge on the surveillance system amongst HCWs due to infrequent training is considered a key factor affecting IDSR implementation [23]. For instance, lack of trained personnel has a direct effect on efficiency of surveillance functions such as laboratory capacity [48]. In addition, improved IDSR system performance was attributed to increased training support

at the regional level in Uganda [48]. Training has a direct effect on various surveillance core functions, for instance limited capacity to undertake data analysis by surveillance focal persons is attributed to inadequate training [30]. Similarly, training can enhance health worker knowledge on surveillance system, data accuracy and improve reporting timeliness and completeness [21, 22, 52]. Eminent disparities were evident in training activities undertaken at lower surveillance levels compared to regional and national levels [35]. Most training providers are at the regional level and rarely are training activities initiated at HF level [23]. On-job training of HP during supervisory visits and sensitisation meetings is the common strategy applied or recommended especially at HF level with limited formal training on IDSR implementation [31, 33, 34]. Training influences health worker's willingness to be involved in surveillance activities [20]. It was significantly associated with easing understanding of surveillance system processes [20]. This corresponds to training impacting health worker's attitudes towards surveillance data reporting and value placed on quality data generation [23]. In addition, training quality has an effect on the knowledge quality gained by HWs translating to an effective surveillance system [23]. The irregular approach to IDSR implementation fails to give due importance to HP training, hence resulting in minimal surveillance system performance [34]. Furthermore, training plays a critical role in identification of appropriate IDSR focal persons, hence influencing reporting quality and accuracy [36, 37]. An identified challenge facing IDSR training is inadequate funding to facilitate formal training activities resulting to on-site orientation of new health staff on need-basis [41].

Frequent supervision influences regular and surveillance data reporting quality by HP [21]. Similarly, adequate supervision ensures reporting is done through the correct channels [33]. Surveillance activities frequent and close supervision would ensure utilisation of current information and materials [35]. Hence, ensuring public health actions are well informed by reliable

surveillance data rather than outdated information. Supervisory activities lack consistency with efforts mostly initiated during disease outbreaks [41]. Limited supervision was observed in lower surveillance levels as opposed to higher levels in Cameroon [35]. Supervisory activities were partially undertaken at the regional levels and hardly executed at HF and community levels [35]. Inconsistent supervisory efforts is a major challenge facing effective IDSR implementation and performance [45]. Factors hindering efficient supervision within surveillance systems involve lack of funding, inadequate transport, limited surveillance personnel and disinterest in surveillance activities [21, 41-43]. Enhanced supervision can be achieved through having well formulated supervisory plans and checklists to monitor surveillance activities [42]. Regular and close supervision motivates health staff involvement in surveillance activities as opposed to viewing IDSR system processes as being cumbersome and interfering with their day-to-day duties [23].

Adequate resource provision facilitates IDSR system optimal functioning. The IDSR strategy was founded on the principle of utilising scarce healthcare resources to effectively achieve disease prevention and control. For instance, reporting forms unavailability may undermine surveillance data quality resulting from incomplete and missing data [53]. Unavailability of surveillance data management tools in the form of computers and calculators especially at HF levels limits surveillance data analysis [35]. Similarly, lack of technical guidelines especially at HF levels has a negative impact on IDSR implementation [41]. Most health systems in Africa especially at peripheral levels rely entirely on paper-based reporting mechanisms due to limited technological and infrastructural capacity [22]. In addition, effective disease surveillance is difficult to achieve in a health system with inadequate infrastructure and limited HP encumbered with surveillance data reporting processes [21]. Moreover, frequent trained health staff turnover has a limiting effect on IDSR system optimal functioning [21].

The main factors contributing to low quality surveillance data generation are attributed to inadequate funding, limited human resource capacity and unavailability of supporting materials [32, 53]. Advancements achieved with IDSR system indicators are linked to sustained financial support, hence resulting in IDSR performance stable progression [48]. Moreover, sustained funding support is vital to strengthening other vital surveillance functions inclusive of training activities on IDSR system [34]. Availing supporting surveillance materials including technical guidelines and reporting forms improves data quality. However, reports indicated IDSR technical guidelines were hardly availed by HWs in health facilities upon request [35].

In the pre-adoption phase of revised IDSR guidelines, sensitization and HP training would aid improved reporting timeliness and completeness [54, 55]. Similarly, post-adopting the revised IDSR guidelines identified enhanced health worker training as a strategy for improved reporting completeness and timeliness [44, 45]. Infrastructural constraints relating to logistical and communication systems negatively impact reporting timeliness [56]. Hence, calls for designing and adapting electronic or mobile reporting systems are justified [19, 20, 31, 43, 44, 57, 58]. Unavailability of specific dates for disease notification from HFs to the next surveillance levels hindered efforts to ascertain reporting timeliness prior to adopting revised IDSR guidelines [55]. This systemic challenge was still persistent even in the periods following revitalised IDSR system adoption [30, 31, 39]. Electronic data transfer methods have a direct effect on surveillance data quality [59]. Furthermore, incomplete and unstandardised physical records in HF registers compromise data accuracy and reliability [9]. Quality control systems incorporation would improve existing surveillance systems' structural capacity to ensure data quality and accuracy are achieved [9].

Evidently, HWs in Madagascar were more aware of case definitions for common conditions such as malaria, diarrhea and respiratory infections compared to other neglected conditions like dengue fever [44]. This low awareness resulted from lack of case definition guidelines, terms of reference and inadequate IDSR training, hence influencing surveillance system's simplicity and applicability to other neglected conditions [21, 44, 60]. Moreover, effective disease surveillance systems performance depends on ease of understanding system's functionalities [31, 33, 39, 61]. Complex reporting systems tend to demoralise health staff from fully participating in case reporting [31]. Perceived surveillance data use in Zimbabwe was mostly intended for resource mobilisation, planning purposes and to a lesser extent for research purposes [33]. On the other hand, perceived surveillance data usefulness was lower amongst healthcare stakeholders responsible for disease detection and response in comparison to those in health management overseeing surveillance activities [20]. Furthermore, timely distribution of information to policy makers and program implementers would be vital to surveillance data usefulness [30]. An acceptable surveillance and response system is well defined by HCWs willingness to voluntarily participate in surveillance activities [14]. Clarity in the designation of HP responsible for surveillance data reporting is pertinent to ensuring reporting accuracy and quality control [39]. Additionally, surveillance system stability is dependent on specific factors, which form the bearing of effectively executing surveillance activities. The functioning state of surveillance core and support functions for instance case confirmation, training, human resources, equipment and communication infrastructure may influence surveillance system's stability [31, 33, 39-41].

Adopting mobile technology for surveillance data reporting as opposed to paper-based reporting systems in Africa would ensure speedy data transmission and cost effectiveness [20, 31, 33, 35,

43]. Mobile phones utilisation to facilitate electronic disease notification would lessen the time and cost burden associated with transporting reporting forms to the next level [31]. Efforts for active case searching at peripheral levels can be enhanced through establishing well-structured community based disease surveillance systems [34]. Furthermore, effective active case searching can be achieved through properly designed operational plans targeting priority surveillance areas with high disease reporting sites [42]. Further assessment studies are required in remote settings to strengthen the IDSR system at the peripheral levels bearing higher disease burdens [36]. Similarly, extensive research can identify factors influencing reporting accuracy and inconsistency within surveillance systems [22]. Research efforts initiation to address key challenges affecting IDSR system implementation will ensure surveillance system core, support and attribute functions optimal performance in Africa.

Conclusion

Evidently from this review, consolidated efforts to strengthen all strategic IDSR components is cardinal to achieving effective IDSR strategy implementation in Africa [48]. Key themes derivation along with their frequency and intensity effect sizes will inform efforts of prioritising scarce resources to improve surveillance functions vital to achieving disease elimination. The findings will inform undertaking follow-up primary studies to assess IDSR system core, support and attribute functions relating to Neglected Tropical Diseases (NTDs). Diseases targeted for elimination and eradication within the IDSR system including NTDs, were evidently accorded limited attention in surveillance assessment studies included in the review. Consequently, outcomes from both the systematic review and primary studies with a focus on NTDs and based on health workers' perspectives will inform development of a recommendation-based framework for adoption at the sub-national level in NTDs-endemic countries. Framework validation will

involve key actors across all health system levels to assess feasibility of implementing recommendations to improve NTDs surveillance and ensure acceptability amongst IDSR system users.

In conclusion, apparently most disease surveillance assessment studies conducted in Africa mainly advocated for health worker training [20, 22-27, 31, 33-37, 41, 45]. Training supports and cuts across strengthening all surveillance functions. Moreover, knowledge impartment through training on IDSR system functions was considered a critical strategy to ensure disease surveillance system effective functioning. The review identified feedback and reporting as essential surveillance core functions while training, resources and supervision as vital surveillance support functions as perceived by HCWs in Africa. The predominant recommendations regarding surveillance attributes focused on timeliness and completeness. Hence, health policy reviews with a keen focus on strengthening surveillance reporting, feedback, supervision, health worker training, resources and reporting timeliness and completeness would be necessary to achieve effective IDSR system implementation especially at lower surveillance levels. Furthermore, this review depicts systematic reviews as being pragmatic tools to identifying key health system components requiring strengthening. Therefore, it would be pertinent for the WHO Regional Office for Africa to set-aside ample budgets and constitute health systems review teams to periodically assess surveillance functions performance based on findings emanating from previous surveillance assessment studies to continuously improve IDSR system implementation. This will accurately inform evidence-based efforts to achieving health system strengthening.

Acknowledgement

We acknowledge Mrs. Estelle Grobler (Information Specialist) in the Faculty of Health Sciences Library, University of Pretoria for her assistance in the literature search exercise.

Author Contributions

AKSN: conceived the research idea, developed the proposal, identified the relevant articles for inclusion in the review and screened the titles and abstracts prior to full-text retrieval, quality appraisal and writing the manuscript.

RCK: identified additional articles for inclusion, screening of titles and abstracts, quality appraisal and writing the manuscript.

CMM and KV: provided supervision, intellectual input and guidance during proposal development and writing the manuscript.

All the authors read and approved the final version of the manuscript before submission.

References

1. Thacker SB, Berkelman RL. Public health surveillance in the United States. Epidemiol Rev. 1988;10:164-90.
2. WHO. Everybody's business: Strengthening health systems to improve health outcomes: WHO's framework for action. Geneva, Switzerland: World Health Organization; 2007.
3. WHO. International Health Regulations (2005): World Health Organization; 2008.
4. Lukwago L, Nanyunja M, Ndayimirije N, Wamala J, Malimbo M, Mbabazi W, et al. The implementation of Integrated Disease Surveillance and Response in Uganda: a review of progress and challenges between 2001 and 2007. Health Policy Plan. 2013;28(1):30-40.
5. WHO. Technical guidelines for integrated disease surveillance and response in the African region. Brazzaville, Republic of Congo and Atlanta, USA. 2010:1-398.
6. Kasolo F, Yoti Z, Bakyaita N, Gaturuku P, Katz R, Fischer JE, et al. IDSR as a Platform for Implementing IHR in African Countries. Biosecurity and Bioterrorism : Biodefense Strategy, Practice, and Science. 2013;11(3):163-9. doi: 10.1089/bsp.2013.0032.
7. Chretien J-P, Lewis SH, editors. Electronic public health surveillance in developing settings: meeting summary. BMC Proc; 2008: BioMed Central.
8. WHO. Integrated Disease Surveillance Quarterly Bulletin. 2016.
9. Phalkey RK, Yamamoto S, Awate P, Marx M. Challenges with the implementation of an Integrated Disease Surveillance and Response (IDSR) system: systematic review of the lessons learned. Health Policy Plan. 2015;30(1):131-43. doi: 10.1093/heapol/czt097 [doi].
10. Sahal N, Reintjes R, Aro AR. Communicable diseases surveillance lessons learned from developed and developing countries: Literature review. Scand J Soc Med. 2009;37(2):187-200.
11. Riesenberg LA, Justice EM. Conducting a successful systematic review of the literature, part 1. Nursing2019. 2014;44(4):13-7.
12. Stern C, Jordan Z, McArthur A. Developing the review question and inclusion criteria. AJN The American Journal of Nursing. 2014;114(4):53-6.
13. WHO A. Assessment protocol for national disease surveillance systems and epidemic preparedness and response. Harare, Zimbabwe: WHO Regional Office for Africa, 2000 Contract No.: Report.
14. German RR, Lee LM, Horan JM, Milstein R, Pertowski C, Waller M. Updated guidelines for evaluating public health surveillance systems. MMWR Recomm Rep. 2001;50(1-35).
15. Moher D, Liberati A, Tetzlaff J, Altman DG. Preferred reporting items for systematic reviews and meta-analyses: the PRISMA statement. Ann Intern Med. 2009;151(4):264-9.
16. Popay J, Roberts H, Sowden A, Petticrew M, Arai L, Rodgers M, et al. Guidance on the conduct of narrative synthesis in systematic reviews. A product from the ESRC methods programme Version. 2006;1:b92.
17. Thomas J, Harden A. Methods for the thematic synthesis of qualitative research in systematic reviews. BMC Med Res Methodol. 2008;8(1):45.
18. Dang D, Dearholt SL. Johns Hopkins nursing evidence-based practice: Model and guidelines: Sigma Theta Tau; 2017.
19. Dairo M, Afolayan D, Akinyemi M. Compliance with epidemic-prone diseases surveillance and response guidelines among health officers at surveillance units in South-West Nigeria. Afr Health Sci. 2018;18(2):428-36. doi: https://dx.doi.org/10.4314/ahs.v18i2.28.

20. Benson FG, Musekiwa A, Blumberg L, Rispel LC. Survey of the perceptions of key stakeholders on the attributes of the South African Notifiable Diseases Surveillance System. BMC Public Health. 2016;16(1):1120. doi: 10.1186/s12889-016-3781-7 [doi].
21. Adokiya MN, Awoonor-Williams JK, Barau IY, Beiersmann C, Mueller O. Evaluation of the integrated disease surveillance and response system for infectious diseases control in Northern Ghana. BMC Public Health. 2015;15:75-015-1397-y. doi: 10.1186/s12889-015-1397-y [doi].
22. Adokiya MN, Awoonor-Williams J, Beiersmann C, Mueller O. Evaluation of the reporting completeness and timeliness of the integrated disease surveillance and response system in northern Ghana. Ghana Med J. 2016;50(1):3-8.
23. Haakonde T, Lingenda G, Munsanje F, Chishimba K. Assessment of Factors Affecting the Implementation of the Integrated Disease Surveillance and Response in Public Health Care Facilities-The Case of Rufunsa District, Zambia. 2018.
24. Lar LA, Afolaranmi TO, Tagurum YO, Uzochukwu B, Zoakah AI. Challenges of integrated disease surveillance response reporting among healthcare personnel in Mangu, Plateau State, Nigeria. Journal of Public Health and Epidemiology. 2015;7(4):108-13.
25. Iwu AC, Diwe KC, Merenu IA, Duru CB, Uwakwe KA. Assessment of disease reporting among health care workers in a South Eastern State, Nigeria. International Journal Of Community Medicine And Public Health. 2016;3(10):2766-74.
26. Nnebue C, Onwasigwe C, Adogu P, Adinma E. Challenges of data collection and disease notification in Anambra State, Nigeria. Tropical Journal of Medical Research. 2014;17(1):1-6. doi: 10.4103/1119-0388.130173.
27. Nnebue C, Onwasigwe C, Ibeh C, Adogu P. Effectiveness of data collection and information transmission process for disease notification in Anambra State, Nigeria. Niger J Clin Pract. 2013;16(4).
28. Begashaw B, Tesfaye T. Assessment of integrated disease surveillance and response implementation in special health facilities of Dawuro Zone. J Anesthesiol. 2016;4(311):11-5.
29. Abubakar A, Sambo M, Idris S, Sabitu K, Nguku P. Assessment of integrated disease surveillance and response strategy implementation in selected Local Government Areas of Kaduna state. 2013. p. 14-9.
30. Mboera LEG, Rumisha SF, Mlacha T, Mayala BK, Bwana VM, Shayo EH. Malaria surveillance and use of evidence in planning and decision making in Kilosa District, Tanzania. Tanzania journal of health research. 2017;19(3). doi: 10.4314/thrb.v19i3.7.
31. Maponga BA, Chirundu D, Shambira G, Gombe NT, Tshimanga M, Bangure D. Evaluation of the Notifiable diseases surveillance system in Sanyati district, Zimbabwe, 2010-2011. The Pan African medical journal. 2014;19:278. doi: 10.11604/pamj.2014.19.278.5202 [doi].
32. Adokiya MN, Awoonor-Williams JK, Beiersmann C, Muller O. The integrated disease surveillance and response system in northern Ghana: challenges to the core and support functions. BMC Health Serv Res. 2015;15(Generic):288. Epub 2015/07/29. doi: 10.1186/s12913-015-0960-7. PubMed PMID: 26216356; PubMed Central PMCID: PMCPMC4515924.
33. Tsitsi JP, Nomagugu N, Gombe NT, Tshimanga M, Donewell B, Mungati M, et al. Evaluation of the Notifiable Diseases Surveillance System in Beitbridge District, Zimbabwe 2015. Open Journal of Epidemiology. 2015;5(03):197.
34. Issah K, Nartey K, Amoah R, Bachan EG, Aleeba J, Yeetey E, et al. Assessment of the usefulness of integrated disease surveillance and response on suspected ebola cases in the

Brong Ahafo Region, Ghana. Infectious diseases of poverty. 2015;4(1):17. doi: 10.1186/s40249-015-0051-3.
35. Ngwa MC, Liang S, Mbam LM, Mouhaman A, Teboh A, Brekmo K, et al. Cholera public health surveillance in the Republic of Cameroon-opportunities and challenges. Pan Afr Med J. 2016;24:222-. doi: 10.11604/pamj.2016.24.222.8045.
36. Mwatondo AJ, Ng'ang'a Z, Maina C, Makayotto L, Mwangi M, Njeru I, et al. Factors associated with adequate weekly reporting for disease surveillance data among health facilities in Nairobi County, Kenya, 2013. The Pan African medical journal. 2016;23:165. doi: 10.11604/pamj.2016.23.165.8758.
37. Baghdadi I. Assessment of core and support functions of case-based surveillance of meningitis in hospitals in Khartoum State in 2015/Evaluation des fonctions essentielles et des fonctions d'appui du systeme de surveillance des cas de meningite dans les hopitaux de l'Etat de Khartoum en 2015. Eastern Mediterranean Health Journal. 2016;22(4):280.
38. Adokiya MN, Awoonor-Williams JK. Ebola virus disease surveillance and response preparedness in northern Ghana. Global health action. 2016;9(1). doi: 10.3402/gha.v9.29763.
39. Mairosi N, Tshuma C, Juru T, Gombe N, Shambira G, Tshimanga M. Evaluation of Notifiable Disease Surveillance System in Centenary District, Zimbabwe, 2016. Open Journal of Epidemiology. 2017;7(03):251.
40. Benson FG, Musekiwa A, Blumberg L, Rispel LC. Comparing laboratory surveillance with the notifiable diseases surveillance system in South Africa. Int J Infect Dis. 2017;59:141-7. Epub 2017/05/24. doi: 10.1016/j.ijid.2017.03.007. PubMed PMID: 28532981.
41. Mandyata CB, Olowski LK, Mutale W. Challenges of implementing the integrated disease surveillance and response strategy in Zambia: A health worker perspective. BMC Public Health. 2017;17(1). doi: 10.1186/s12889-017-4791-9.
42. Lakew GA, Wassie E, Ademe A, Fenta A, Wube S, Werede M, et al. Status of surveillance and routine immunization performances in Amhara Region, Ethiopia: findings from in-depth peer review. The Pan African medical journal. 2017;27:6. doi: 10.11604/pamj.supp.2017.27.2.10755.
43. Wu TSJ, Kagoli M, Kaasboll JJ, Bjune GA. Integrated Disease Surveillance and Response (IDSR) in Malawi: Implementation gaps and challenges for timely alert. PLoS One. 2018;13(11). doi: 10.1371/journal.pone.0200858. PubMed PMID: WOS:000451763800004.
44. Randriamiarana R, Raminosoa G, Vonjitsara N, Randrianasolo R, Rasamoelina H, Razafimandimby H, et al. Evaluation of the reinforced integrated disease surveillance and response strategy using short message service data transmission in two southern regions of Madagascar, 2014-15. BMC Health Serv Res. 2018;18(1):265. Epub 2018/04/11. doi: 10.1186/s12913-018-3081-2. PubMed PMID: 29631631; PubMed Central PMCID: PMCPMC5891931.
45. Masiira B, Nakiire L, Kihembo C, Katushabe E, Natseri N, Nabukenya I, et al. Evaluation of integrated disease surveillance and response (IDSR) core and support functions after the revitalisation of IDSR in Uganda from 2012 to 2016. BMC Public Health. 2019;19(1):46. Epub 2019/01/11. doi: 10.1186/s12889-018-6336-2. PubMed PMID: 30626358; PubMed Central PMCID: PMCPMC6327465.
46. Yahaya A, Ndihokubwayo J, Coulibaly S, Akanmori B, Mwenda J, Dosseh A. Laboratory capacity in 2012 for diagnosis of epidemic prone diseases in the context of Integrated Disease Surveillance and Response in the WHO African Region. African Health Monitor. 2013;(18):44-8.

47. Dairo M, Bamidele J, Adebimpe W. Disease surveillance and reporting in two Southwestern states in Nigeria: Logistic challenges and prospects. Journal of public health and Epidemiology. 2010;2(6):125-9.
48. Lukwago L, Nanyunja M, Ndayimirije N, Wamala J, Malimbo M, Mbabazi W, et al. The implementation of Integrated Disease Surveillance and Response in Uganda: A review of progress and challenges between 2001 and 2007. Health Policy Plan. 2013;28(1):30-40. doi: 10.1093/heapol/czs022.
49. Mboera L, Rumisha S, Magesa S, Kitua A. Utilisation of health management information system in disease surveillance in Tanzania. Tanzania journal of health research. 2001;3(2):15-7.
50. Mghamba JM, Mboera LEG, Krekamoo W, Senkoro KP, Rumisha SF, Shayo E, et al. Challenges of implementing an integrated disease surveillance and response strategy using the current health management information system in Tanzania. Tanzania journal of health research. 2004;6(2):57-63.
51. Gueye D, Senkoro KP, Rumisha SF. Baseline monitoring and evaluation of integrated disease surveillance and response in Tanzania. 2005.
52. Bawa SB, Olumide EA. The effect of training on the reporting of notifiable diseases among health workers in Yobe State, Nigeria. Niger Postgrad Med J. 2005;12(1):1-5. Epub 2005/04/14. PubMed PMID: 15827587.
53. Phalkey RK, Shukla S, Shardul S, Ashtekar N, Valsa S, Awate P, et al. Assessment of the core and support functions of the Integrated Disease Surveillance system in Maharashtra, India. BMC Public Health. 2013;13(1):575. doi: 10.1186/1471-2458-13-575.
54. Franco LM, Fields R, Mmbuji PK, Posner S, Mboera LE. Situation analysis of infectious disease surveillance in two districts in Tanzania 2002. 2003.
55. Pond B, El Sakka H, Wamala J, Lukwago L. Mid-term evaluation of the integrated disease surveillance and response project. Washington, DC: USAID. 2011.
56. Mboera LE, Rumisha SF, Mwanemile EJ, Mziwanda E, Mmbuji PK. Enhancing disease surveillance reporting using public transport in Dodoma District, Central Tanzania. Tanzan Health Res Bull. 2005;7(3):201-5.
57. Mtema Z, Changalucha J, Cleaveland S, Elias M, Ferguson HM, Halliday JE, et al. Mobile phones as surveillance tools: implementing and evaluating a large-scale intersectoral surveillance system for rabies in Tanzania. PLoS Med. 2016;13(4):e1002002.
58. Kiberu VM, Matovu JK, Makumbi F, Kyozira C, Mukooyo E, Wanyenze RK. Strengthening district-based health reporting through the district health management information software system: the Ugandan experience. BMC Med Inform Decis Mak. 2014;14:40-6947-14-40. doi: 10.1186/1472-6947-14-40 [doi].
59. Awoonor-Williams JK, Bawah AA, Nyonator FK, Asuru R, Oduro A, Ofosu A, et al. The Ghana essential health interventions program: a plausibility trial of the impact of health systems strengthening on maternal & child survival. BMC Health Serv Res. 2013;13(2):S3.
60. Nsubuga P, Brown WG, Groseclose SL, Ahadzie L, Talisuna AO, Mmbuji P, et al. Implementing Integrated Disease Surveillance and Response: Four African countries' experience, 1998-2005. Global public health. 2010;5(4):364-80. doi: 10.1080/17441690903334943 [doi].
61. Mutale W, Chintu N, Amoroso C, Awoonor-Williams K, Phillips J, Baynes C, et al. Improving health information systems for decision making across five sub-Saharan African

countries: implementation strategies from the African Health Initiative. BMC Health Serv Res. 2013;13(2):S9.

Supporting information

Table S1. PRISMA checklist

Figure S1. John Hopkins Nursing Evidence-Based Practice Appraisal Tool

Appendix S1. PubMed search strategy

Appendix S2. PROSPERO protocol

Flow chart summarising the systematic review process

Click here to download Figure Fig 1. Flow Chart summarising the systematic review process.tif

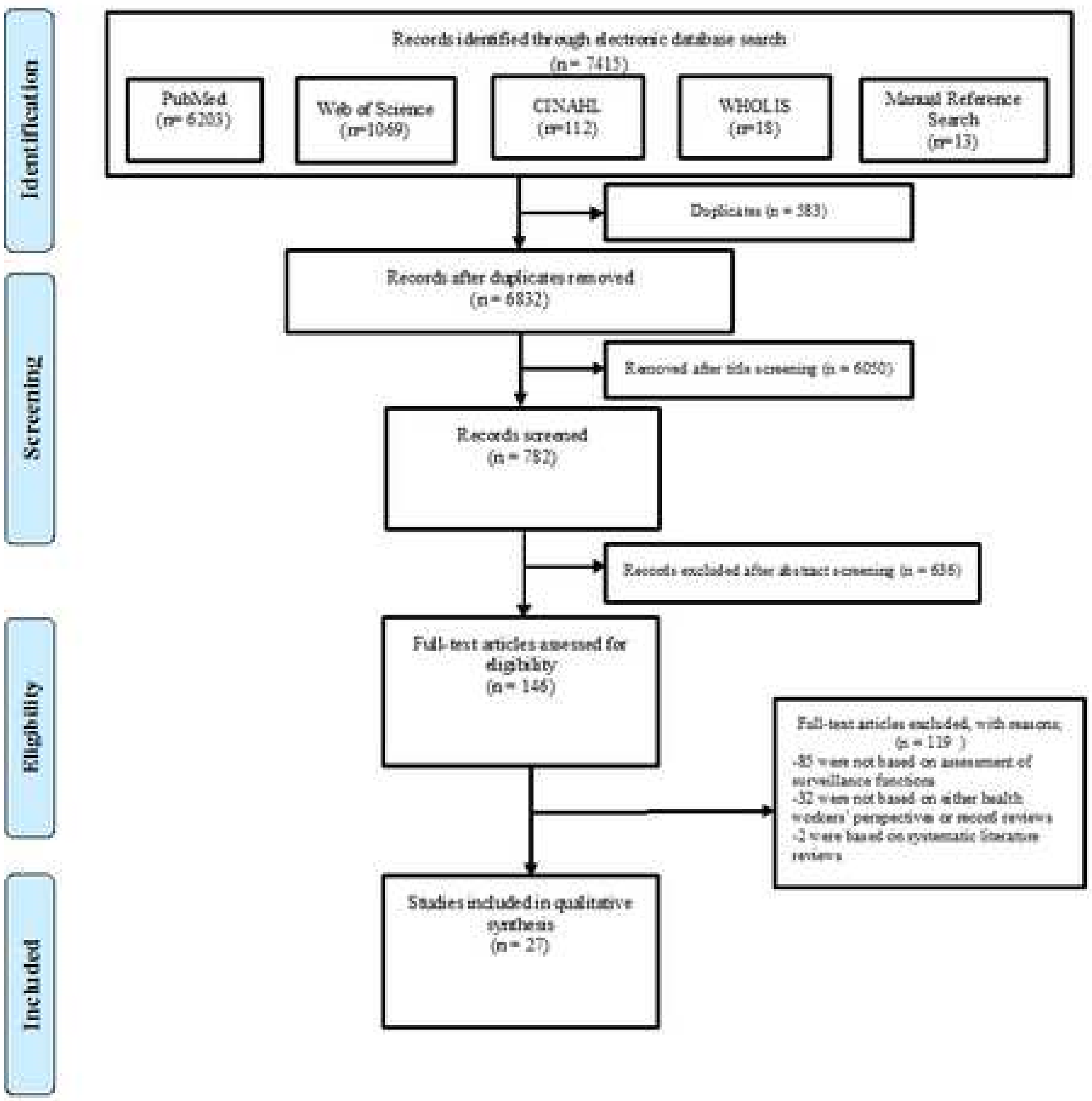

Table S1. PRISMA checklist

Click here to access/download
Supporting Information
Table S1_PRISMA checklist.docx

Figure S1. Johns Hopkins Nursing Evidence-Based Practice Appraisal Tool

Click here to access/download

Supporting Information

Johns Hopkins Nursing Evidence-Based Practice Appraisal Tool.pdf

Appendix S1. PubMed Search Strategy

Click here to access/download
Supporting Information
Appendix S1_PubMed Search Strategy.docx

Click here to access/download
Supporting Information
Appendix S2_PROSPERO protocol_CRD42019124108.pdf
