# Supplementary material for: A systematic review on improving implementation of the revitalised integrated disease surveillance and response system in the African region: A health workers’ perspective
Source: PLoS One. 2021 Mar 19;16(3):e0248998. doi: 10.1371/journal.pone.0248998 (PMC7978283; doi:10.1371/journal.pone.0248998)
Supplement: Attachment — Submitted filename: Response to Reviewers.docx [file pone.0248998.s010.docx]

**Response to academic editor and reviewer comments**

**Manuscript PONE-D-19-27712R2**

**Title:** A systematic review on improving implementation of the revitalised integrated disease surveillance and response system in the African region: a health workers' perspective”

**Authors:**

Arthur Kipkemoi Saitabau Ng'etich ([arthursaitabau@yahoo.com](mailto:arthursaitabau@yahoo.com))

Kuku Voyi ([kuku.voyi@up.ac.za](mailto:kuku.voyi@up.ac.za))

Ruth Kirinyet ([kirinyet.ruth@ku.ac.ke](mailto:kirinyet.ruth@ku.ac.ke))

Clifford Maina Mutero ([cmutero@icipe.org](mailto:cmutero@icipe.org))

Dear Editor,

Thank you for giving us the opportunity to re-submit a revised version of our manuscript for publication consideration in *PLOS One* journal. We appreciate the time and effort dedicated by the academic editor and reviewers to provide insightful feedback on the revised manuscript. We have revised the manuscript considering all the comments provided. Please find below a point-by-point response to the comments. All page and line numbers cited herein refer to the revised manuscript (without track changes).

**Academic Editor:**

**Journal Requirements:**

Please review your reference list to ensure that it is complete and correct. If you have cited papers that have been retracted, please include the rationale for doing so in the manuscript text, or remove these references and replace them with relevant current references. Any changes to the reference list should be mentioned in the rebuttal letter that accompanies your revised manuscript. If you need to cite a retracted article, indicate the article’s retracted status in the References list and also include a citation and full reference for the retraction notice.

**Author response:** The reference list has been reviewed to ensure all references are complete and correct. However, two references (refer below) in the previous manuscript were replaced with a relevant and updated reference (References, line 879-881, page 54). We further checked all references and can confirm to the best of our knowledge that none of the articles cited were retracted.

*-Franco LM, Fields R, Mmbuji PK, Posner S, Mboera LE. Situation analysis of infectious disease surveillance in two districts in Tanzania 2002. Working Paper; 2003.*

*-Pond B, El Sakka H, Wamala J, Lukwago L. Mid-term evaluation of the integrated disease surveillance and response project. Washington, DC: USAID. 2011.*

**Reviewers’ comments:**

1. If the authors have adequately addressed your comments raised in a previous round of review and you feel that this manuscript is now acceptable for publication, you may indicate that here to bypass the “Comments to the Author” section, enter your conflict of interest statement in the “Confidential to Editor” section, and submit your "Accept" recommendation.

Reviewer #3: All comments have been addressed

Reviewer #4: (No Response)

**Author:** No Response

2. Is the manuscript technically sound, and do the data support the conclusions?

The manuscript must describe a technically sound piece of scientific research with data that supports the conclusions. Experiments must have been conducted rigorously, with appropriate controls, replication, and sample sizes. The conclusions must be drawn appropriately based on the data presented.

Reviewer #3: Yes

Reviewer #4: Yes

**Author:** No Response

3. Has the statistical analysis been performed appropriately and rigorously?

Reviewer #3: Yes

Reviewer #4: Yes

**Author:** No Response

4. Have the authors made all data underlying the findings in their manuscript fully available?

The [PLOS Data policy](http://www.plosone.org/static/policies.action#sharing) requires authors to make all data underlying the findings described in their manuscript fully available without restriction, with rare exception (please refer to the Data Availability Statement in the manuscript PDF file). The data should be provided as part of the manuscript or its supporting information, or deposited to a public repository. For example, in addition to summary statistics, the data points behind means, medians and variance measures should be available. If there are restrictions on publicly sharing data—e.g. participant privacy or use of data from a third party—those must be specified.

Reviewer #3: Yes

Reviewer #4: Yes

**Author:** No Response

5. Is the manuscript presented in an intelligible fashion and written in standard English?

PLOS ONE does not copyedit accepted manuscripts, so the language in submitted articles must be clear, correct, and unambiguous. Any typographical or grammatical errors should be corrected at revision, so please note any specific errors here.

Reviewer #3: Yes

Reviewer #4: No

**Author response:** The manuscript has been be revised accordingly by checking and correcting all typographical and grammatical errors.

6. Review Comments to the Author
Please use the space provided to explain your answers to the questions above. You may also include additional comments for the author, including concerns about dual publication, research ethics, or publication ethics. (Please upload your review as an attachment if it exceeds 20,000 characters)

Reviewer #3: (No Response)

Reviewer #4: According to previous reviewers' comments and authors' answers, the manuscript has been critically revised. However, authors should further concise the Table 1 and the conclusion part. In the next round of review, authors should submit a clear vision of manuscript without changing traces.

**Author response:** Table 1 and the conclusion section have been revised as suggested by the reviewer. The final version of the revised manuscript is without any track changes.

All authors reviewed the final version of the revised manuscript and ensured it adheres to all editorial requirements. We hope that our response to the comments provided render the revised manuscript suitable for publication in *PLOS One* journal.

Yours Sincerely,

Arthur Kipkemoi Saitabau Ng’etich

(Corresponding author)
